# Comparative Analysis of Lavandula Dentata Rhizosphere Microbiota Across Different Developmental Stages in a Semi‐Arid Area

**DOI:** 10.1111/1758-2229.70318

**Published:** 2026-03-17

**Authors:** Oumaima Akachoud, Julien Langrand, Hafida Bouamama, Natacha Facon, Frédéric Laruelle, Ahmed Qaddoury, Anissa Lounès ‐Hadj Sahraoui

**Affiliations:** ^1^ Université Littoral Côte D'opale, UCEIV—UR n°4492, Unité de Chimie Environnementale et Interactions Sur le Vivant Calais France; ^2^ Laboratoire d'Agrobiotechnologie et Bioingénierie, Faculté Des Sciences et Techniques (FST) Université Cadi Ayyad Marrakech Morocco; ^3^ Laboratoire de Recherche en Développement Durable et Santé, Faculté Des Sciences et Techniques (FST)—Université Cadi Ayyad Marrakech Morocco

**Keywords:** developmental stages, *Lavandula dentata*, microbial diversity, network interactions, PLFA, soil microbiome

## Abstract

The positive effects of soil microbiota on plant growth and stress tolerance are well established. However, their role in aromatic and medicinal plants, particularly under arid conditions, remains underexplored. This study examined rhizospheric microbial community dynamics across developmental stages of wild *Lavandula dentata* L*.*, a semi‐arid species threatened with extinction in Morocco. Results showed total microbial biomass peaked at senescence, mainly due to increases in Gram‐negative (25.02 μg/g) and Gram‐positive (18.11 μg/g) bacterial biomasses. Beta diversity analysis revealed consistent dominance of Actinobacteria, with peaks during senescence and the vegetative phase. Saprotrophic fungi (8.81 μg/g) and arbuscular mycorrhizal fungi (AMF) (4.16 μg/g) biomasses peaked at flowering. The fungal community was dominated by the Ascomycota phylum, with no significant variation across stages. The AMF genus *Glomus* remained most abundant throughout development. Senescence featured the most complex interkingdom interaction network and high ecological niche heterogeneity, reflected by more negative associations. Overall, the rhizospheric microbial community of *L. dentata* shifts with plant development, with flowering and senescence as key phases for microbial biomass accumulation and community diversification. Flowering and senescienceez stages seem to represent promising targets for developing biostimulant consortia to improve soil health and crop productivity in arid environments.

## Graphical abstract

This study aims to give deeper insight into the relationship between the dynamics of the rhizospheric microbiota and the developmental stages of *L. dentata* growing spontaneously in a semi‐arid environment. To achieve this objective, we examined and monitored the biomass (using phospholipid fatty acid, PLFA analysis), diversity and network complexity of soil bacterial and fungal communities (using a meta‐barcoding approach). Results showed that soil microbial biomass was highest at the senescence stage, mainly driven by an increase in bacterial biomass. Bacterial richness was lowest at the vegetative stage, whereas fungal richness remained stable across developmental stages. Actinobacteriota dominated the bacterial community, and Ascomycota dominated the fungal community at all stages. Furthermore, microbial network analysis revealed increased complexity of soil microbial interactions during senescence.

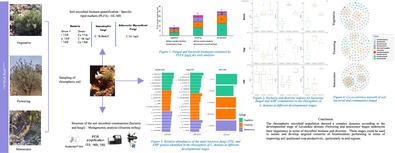

## 1 Introduction

Aromatic and medicinal plants (AMP) have historical significance in human civilizations due to their aroma‐therapeutic‐cosmetic‐culinary applications and several biological properties, thanks to their various bioactive molecules. They have recently gained interest as a sustainable agricultural solution to mitigate microbial diseases during cultivation and post‐harvest, for example, essential oils (EO) of *Mentha pulegium* L., *Origanum elongatum* (Bonnet) Emb. & Maire., *Thymus vulgaris* L. and *Corymbia citriodora* K.D.Hill & L.A.S.Johnson, reduced leaf lesion size by up to 84.2% in vivo in grape berry disease caused by *Botrytis cinerea* Pers., (Ur Rehman et al. 2021; Raveau et al. 2022; Aoujil et al. 2025). Nowadays, improving AMP production is crucial to meet the growing market demand, primarily through the domestication of spontaneous species with high economic value. This requires a good understanding of the relationship between their phytochemical and biological performances, pedoclimatic conditions of their native habitats, and their interactions with soil microbiota (Hamidah et al. 2018).

Soil microbiota, can improve plant growth and tolerance to abiotic and biotic stresses through various mechanisms (Azizi et al. 2021; Israel et al. 2022; Koza et al. 2022). Soil microbiota can also influence the production and concentration of secondary metabolites stimulating host plant biosynthesis and signalling pathways (Pascale et al. 2020; Zhao et al. 2022). Moreover, microorganisms influence plant root exudates which in turn influence the structure of soil microbial communities (Chaparro et al. 2013; Micallef et al. 2009) through their action on the secretion of low and high‐molecular‐weight compounds (Badri et al. 2009). These compounds act as substrates, and signalling molecules, that induce changes in soil microbial composition (Badri et al. 2009, 2013). Root exudates can also alter soil pH and redox potential due to their organic acid composition, further shaping microbial communities (Shi et al. 2011). The quality and quantity of root exudates change with plant species and development stage (Uren 2000; Chaparro et al. 2013), resulting in alteration of microbial communities in the rhizosphere of many plant species, such as rice, mouse‐ear cress, soybean and Drummond's rockcress (Chaparro et al. 2014; Edwards et al. 2015; Dombrowski et al. 2017).

Several AMP species grow in the harsh conditions of arid and semi‐arid regions and are exposed to several abiotic (drought, high temperature, degraded soils, etc.) and anthropogenic (overgrazing, inappropriate harvesting, etc.) constraints, resulting in reduced growth and biomass production, or even endangering overexploited AMP species such as *Lavandula dentata* (Mehdioui and Kahouadji 2007; Fennane and Rejdali 2016). *L. dentata* , belonging to the *Lamiaceae* family, is an aromatic shrub native to northwestern Africa, Spain, Jordan, Ethiopia and the Arabian Peninsula (Rankou et al. 2015; Fennane and Rejdali 2016). It is known for its distinctive serrated leaves and purple spikes. It is valued for its essential oils (EO) rich in terpene compounds (Giuliani et al. 2020) with various biological activities, including antioxidant, antimicrobial, phytotoxic and insecticidal properties (Rahmouni et al. 2019; Wagner et al. 2021). Moreover a recent research showed that the yield and the quality of *L. dentata* EO varied significantly according to developmental stages, showing a greater yields at the flowering and the senescence stage (Akachoud et al. 2024).

Despite the recognised importance of soil microbiota in plant health and stress resilience, little is known about the rhizospheric microbial communities associated with *L. dentata* , particularly under semi‐arid conditions. Understanding how these communities vary across the plant's developmental stages is essential for elucidating the ecological interactions that support its growth and adaptation. Thus, the current study aims at characterising the structure, biomass and diversity of the rhizospheric microbiota of wild *L. dentata* throughout its phenological stages. To achieve this objective, we examined and monitored the microbial biomass (as an indicator of living microbiota using phospholipid fatty acids, PLFA) and network complexity (using meta‐barcoding approach).

By identifying stage‐specific microbial trends and interaction patterns, this work aims to lay the groundwork for future targeted research on the functional roles of key microbial taxa, their influence on secondary metabolite production, and their potential application in sustainable agriculture and the conservation of this threatened aromatic species.

## 2 Materials and Methods

### 2.1 Sampling and Physicochemical Soil Analyses

We collected soil samples, 0–20 cm deep, from the rhizosphere of *L. dentata*, growing spontaneously in the region of Asni, Morocco, (31° 13′ 35.3″ N 7° 57′ 38.2″ W) at the vegetative, flowering and senescence stages. Five plants, spaced at least 150 m apart, were selected to obtain 3 kg of soil (Figure S1).

The studied region under study is characterised by an altitude between 1400 and 1600 m, temperatures ranging from −8°C to 33°C and annual precipitation of 368 mm.

Chemical analyses were performed on dried soil samples (80°C for 24 h). pH (water) and pHKCl (NFX 31–117), total lime (NFX 31–105), total carbon and organic matter (ISO 10‐694), total nitrogen (ISO 13‐878) and assimilable phosphorus (NFX 31–160), as well as exchangeable potassium, calcium and magnesium (NFX 31–160) analyses were performed at the accredited laboratory of the Centre Scientifique Agricole Regional (CESAR), Ceyzeriat, France.

### 2.2 Analysis of Soil Fatty Acids

Soil‐neutral lipid fatty acid (NFLA) and phospholipid fatty acid (PLFA) were determined according to the Frostegård method (Frostegård et al. 1991). Freeze‐dried soil samples (3 g of each) were added to the Blight and Dyer's (BD) extraction solution, which consisted of a mixture of chloroform, methanol and citrate buffer (0.5:1:0.4 v:v:v). The lipid material was fractionated on Solid Phase Extraction (SPE) columns containing silica (6 mL volume, 500 mg sorbent, Interchim, Montluçon, France) by successive elution with chloroform, acetone and methanol (1:2:1, v/v/v) into neutral lipids (NLFA), glycolipids and polar lipids containing phospholipid (PLFA) respectively. The NLFA and PLFA fractions were dried under a stream of N_2_ and saved for fatty acid methyl esters preparations. The NLFA and PLFA fractions were then subjected to mild alkaline methanolysis (0.2 M KOH in methanol). The resulting free fatty acid methyl ester from transesterification were recovered in pure hexane (200 μL) to be analysed using a gas chromatography‐mass spectrometer (GC–MS) Shimadzu QP‐2010 Ultra (Shimadzu, Japan) equipped with a single quadrupole mass spectrometer detector (MS) and simultaneously coupled with a flame ionisation detector (FID). Samples were analysed in split mode (80:1 ratio) on a ZB‐1MS fast capillary column (10 m length × 0.1 mm inner diameter × 0.1 μm phase thickness, 100% dimethylpolysiloxane, Zebron, Phenomenex, Torrance Calif, U.S.A.) using helium as the carrier gas at a constant linear velocity (40 cm sec^−1^). The injector temperature was 280°C, and the detector temperatures were respectively 330°C for FID and 280°C for the ion source.

The temperature program started with an initial temperature of 175°C and increased by 25°C every minute to reach a final temperature of 275°C, which was maintained for 0.5 min. The ionisation mode was electronic impact at 70 eV and the mass range between 50 and 400 u was scanned. The selective ion monitoring (SIM) mode was used simultaneously. Quantification of fatty acids was performed using nonadecanoic acid methyl ester (C19:0, Sigma Aldrich) as an internal standard. Fatty acids were identified by comparing their relative retention time with that of commercial fatty acid methyl ester standards (47080‐U Bacterial Acid Methyl Ester (BAME) Mix, Sigma Aldrich) and comparing their spectra with spectra either obtained from commercial standards and/or published in the literature (NIST Standard Reference Database). Phospholipid fatty acid (PLFA) i15:0, a15:0, i16:0, i17:0, a17:0 and PLFA cy17:0, C18:1ω7, cy19:0 as indicators of Gram‐positive and negative bacterial biomasses, respectively.

### 2.3 DNA Extraction, Amplification and Sequencing

According to the manufacturer's instructions, genomic DNA was extracted from 250 mg of soil samples using the Nucleospin Soil kit (Macherey‐Nagel, Düren, Germany). The purified DNA was filtered and eluted, and the quality of the DNA obtained was assessed using a SpectraMax R iD3 spectrophotometer (Molecular Devices LLC, Sunnyvale, CA, United States) to ensure its purity and concentration.

Two PCR reactions were meticulously performed to amplify each microbial barcode (i.e., bacterial 16S, fungal DNA and AM fungal 18S) using a thermal cycler (Agilent Surecycler 8800, Les Ulis, France). The reaction mixture (25 μL) contained 0.2 mM dNTP, 0.4 μM of each primer, 1 μL DMSO, 100 μg/mL bovine serum albumin, 1 ng gDNA, 0.5 U of Q5 high‐fidelity DNA polymerase (New England Biolabs France, Evry) and its reaction buffer.

For bacterial 16S rRNA amplification, the primer pair 341F/805R (Herlemann et al. 2011) was used. The sequence of the primers with adapters CS1 and CS2 shown in bold were as follows: CS1_341‐F (5′‐**ACA CTG ACG ACA TGG TTC TAC A**CC TAC GGG NGG CWG CAG‐3′), and CS2_805‐R (5′‐**TAC GGT AGC AGA GAC TTG GTC TCT** GAC TACC AGG GTA TCT AAT C‐3′). PCR conditions consisted of an initial denaturation at 95°C for 3 min, followed by 35 cycles of denaturation at 95°C for 30 s, annealing at 56°C for 30 s, and extension at 72°C for 50 s, and a final extension step at 72°C for 5 min. The expected size of the PCR amplicons was approximately 400 bp.

For fungal rDNA amplification, the primer pair KYO2‐F/ITS4 KYO3‐R was used (Toju et al. 2012). The sequence of the primers with adapters CS1 and CS2 shown in bold were as follows: CS1_ITS3‐KYO2‐F (5′‐**ACA CTG ACG ACA TGG TTC TAC A**GA TGA AGA ACG YAG YRA A‐3′), CS2_ITS4‐KYO3‐R (5′‐**TAC GGT AGC AGA GAC TTG GTC TCT** BTT VCC KCT TCA CTC G‐3′). PCR conditions, adapted from Toju et al. (2012), consisted of an initial denaturation at 95°C for 10 min, followed by 35 cycles of denaturation at 94°C for 20 s, annealing at 47°C for 30 s, and extension at 72°C for 20 s, and a final extension step at 72°C for 7 min. The expected size of the PCR amplicons was approximately 327 bp.

A nested PCR was performed to amplify AM fungal 18S rDNA. For the first round of PCR, the primer pair AML1/AML2 was used (Lee et al. 2008). Amplicons were reamplified in a second round PCR using the primers nu‐SSU‐0595/nu‐SSU‐0948 as in Stefani et al. (2020). The sequence of the primers with adapters CS1 and CS2 shown in bold were as follows: CS1_nu‐SSU‐0595‐5‐F (5′‐**ACA CTG ACG ACA TGG TTC TAC A**CG GTA ATT CCA GCT CCA ATA G‐3′), CS2_nu‐SSU‐0948‐3‐R (5′‐**TAC GGT AGC AGA GAC TTG GTC T**TT GAT TAA TGA AAA CAT CCT TGG C‐3′). The first round of PCR conditions, adapted from Lee et al. (2008), consisted of an initial denaturation at 94°C for 3 min, followed by 35 cycles of denaturation at 94°C for 1 min, annealing at 45°C for 1 min and extension at 72°C for 1 min and a final extension step at 72°C for 5 min. For the second round PCR, adapted from Stefani et al. (2020), the thermocycling conditions were the same as for the first round of PCR except for the annealing temperature, which was 50°C. Amplicons were visualised onto 1% agarose gel. The expected size of the PCR amplicons from the second round of PCR was approximately 400 bp (Stefani et al. 2020).

The 16S, 18S and ITS amplicons were shipped to the Genome Quebec Innovation Centre (Montreal, QC, Canada) for library preparation and sequencing. Paired‐end sequencing (2 × 300 bp) was performed using the Illumina MiSeq sequencer at the Genome Quebec Innovation Center. This technique is based on bridge amplification and incorporates reversible terminators labelled with fluorophores, enabling the identification of nucleotide bases and DNA sequencing. The dataset's 16S, ITS and 18S gene sequences have been deposited in the NCBI Sequence Read Archive (SRA) database, which can be found under the accession number (PRJNA943221).

### 2.4 Bioinformatic Analyses

Illumina sequence reads were processed using FROGS (Find Rapidly Out with Galaxy Solution). Sequences containing ambiguous bases (N) or lacking the specific primers were excluded. The Cutadapt software (Martin 2011) was employed to identify and remove primer sequences with less than a 10% difference. A total of 1,103,326, 346,580 and 244,961 sequences were identified for 16S, ITS and 18S, respectively, and the sequence clustering was carried out using the SWARM algorithm (v2.1.5) (Mahé et al. 2014). A preliminary denoising step was conducted to construct highly refined clusters with minimal dissimilarities (*d* = 1), followed by a second step using an aggregation distance of 3. The representative sequences obtained for each cluster, known as Operational Taxonomic Units (OTUs), underwent chimera detection using the VSEARCH algorithm (Rognes et al. 2016) and were removed, along with those having an abundance of less than 0.001% of the total abundance. Ultimately, 12,511, 2141 and 17,998 OTUs were preserved for 16S, ITS and 18S, respectively, which accounted for 197,044, 83,975 and 1,158,241 sequences, respectively. The taxonomic classification of each OTU was performed using the RDP Classifier (Wang et al. 2007) against the Silva database (v138.1), Unite fungi (v8.3) and Silva (v138.1) for 16S, ITS and 18S, respectively. Indeterminate OTUs were affiliated with the taxonomic rank with the highest similarity score using the online Microbial Nucleotide BLAST (BLASTn) program in the NCBI database. The rarefaction and the analyses of richness and diversity were performed using *R* package version R4.1.2. The function assignment taxonomy was performed to assign taxonomy using the reference FUNGUILD database for fungal communities and the FARPOTAX database for bacterial communities.

### 2.5 Statistical Analyses

Statistical analyses were performed by XLSTAT 2022.1.1 (Addinosoft), and statistical significance was analysed by ANOVA completed by a Tukey HSD and Fisher test. Principal component analysis (PCA) based on Pearson's correlation matrix was performed with XLSTAT. Richness (Chao1) and diversity (Simpson, Shannon) indices, PERMANOVA were conducted with the help of the Vegan package in R.

## 3 Results

### 3.1 Soil Physicochemical Analysis and Microbial Biomass Quantification

All soil samples had alkaline pH ranging from 7.98 to 8.08, with high cation exchange capacity (152.39–197.68 meq/kg) and high levels of total carbonates (214–280 g/kg). Soil samples were also rich in organic matter (OM ranging from 24.34 to 79.39 g/kg), with a relatively high C/N ratio (ranging from 19 to 22), high concentration of potassium (0.32 g/kg), calcium (11.18 g/kg) and magnesium (0.46 g/kg), and a very low level of available phosphorus (ranging from 0.007 to 0.01 g/kg) (Table S1).

**FIGURE 1:**
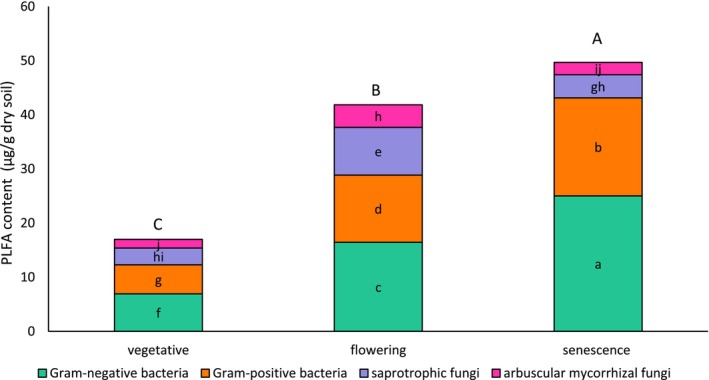
Fungal and bacterial biomasses estimated by PLFA (μg/g dry soil) analysis in the rizosphere of *L. dentata* at different developmental stages. Different capital and lowercase letters indicate significant differences, respectively in the total PLFA and bacterial and fungal biomass, according to Tukey's HSD test (*p* < 0.05).

The total microbial biomass as well as the specific microbial biomass contents of the soil samples varied significantly (*p* < 0.0001) depending on the developmental stage of *L. dentata* (vegetative, flowering and senescence). Gram‐negative bacteria showed the highest biomass, followed by Gram‐positive bacteria, saprotrophic fungi and AMF. The highest PLFA content (49.67 μg/g soil) was recorded at senescence, and the lowest at the vegetative stage (16.97 μg/g) (Figure 1). The variation of bacterial biomass (Gram‐negative and Gram‐positive) showed a similar trend to that of total microbial biomass, with the lowest content at the vegetative stage (12.30 μg/g) and the highest level at senescence (43.13 μg/g). Saprotrophic fungi and AMF biomasses were higher at flowering than at other stages. However, no significant differences were observed between the fungal biomass values recorded at senescence and vegetative stages.

### 3.2 Microbial Diversity and Richness in *L. dentata* Rhizosphereacross Developmental Stages

The Shannon, Chao1 and Simpson diversity indexes and α‐diversity for the bacteria, total fungi and AMF communities were evaluated based on the relative abundance of the microbial species.

**FIGURE 2:**
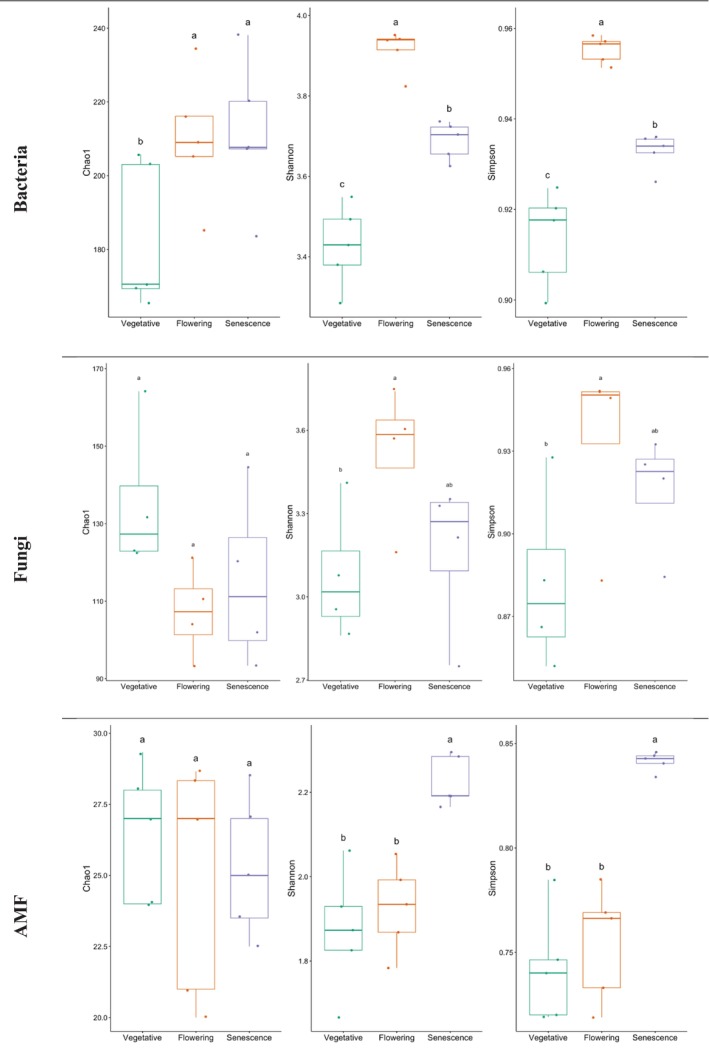
Richness and diversity indexes for bacterial, fungal, and AMF communities in the rhizosphere of *L. dentata* at different developmental stages. Different letters indicate significant differences according to ANOVA test (*p* < 0.05).

According to the Shannon index, the bacterial species diversity was significantly higher at flowering (3.91) than at senescence (3.68) and vegetative (3.42) stages (Figure 1). The Simpson index aligned with the Shannon index at different *L. dentata* 's development stages (Figure 2).

The diversity of fungal species increased at flowering compared to other development stages according to Shannon (3.51) and Simpson (0.93) indexes.

The AMF species diversity was higher at the senescence stage with Shannon, and Simpson indexes values of 2.22 and 0.84 respectively. However, no significant differences were noticed between the vegetative and flowering stages according to both indexes (Figure 2).

In addition, according to Chao1 index, species richness for bacteria, saprotroophic fungi and AMF in the rhizosphere of lavender did not vary significantly between the three development stages (Figure 2).

### 3.3 Microbial Communities' Structure vs. *L. dentata* 's Developmental Stages

In total, 728, 743 and 823 bacterial OTUs were obtained at the vegetative, flowering and senescence stages respectively (Figure S2). The highest number of OTUs was identified at senescence and the lowest at the vegetative stage. Some 494 bacterial OTUs were common to the different development stages. Whereas flowering and senescence stages shared the highest number of OTUs (108), whereas only 60 OTUs were shared between the vegetative and flowering stages (Figure S2).

Concerning the total fungal community, the number of fungal OTUs in the rhizosphere of *L. dentata* varied significantly between its development stages (Figure S2). The highest number of OTUs was identified at senescence (232) and the lowest at flowering (199). About 122 OTUs were common to the different development stages. Although the vegetative and senescence stages showed the highest number of common OTUs (40), the vegetative and flowering stages shared only 23 OTUs (Figure S2).

Regarding the AMF community, the rhizospheric soil of *L. dentata* exhibited a significantly higher number of AMF OTUs at flowering (54) than at senescence (48) and vegetative (43) stages. The number of OTUs common to the different development stages was 37. Flowering and senescence showed the highest number of common OTUs (Figure S2).

### 3.4 Taxonomic and Functional Profiles of *L. dentata* Rhizosphere Microbiota across Developmental Stages

#### 3.4.1 Bacteria

**FIGURE 3:**
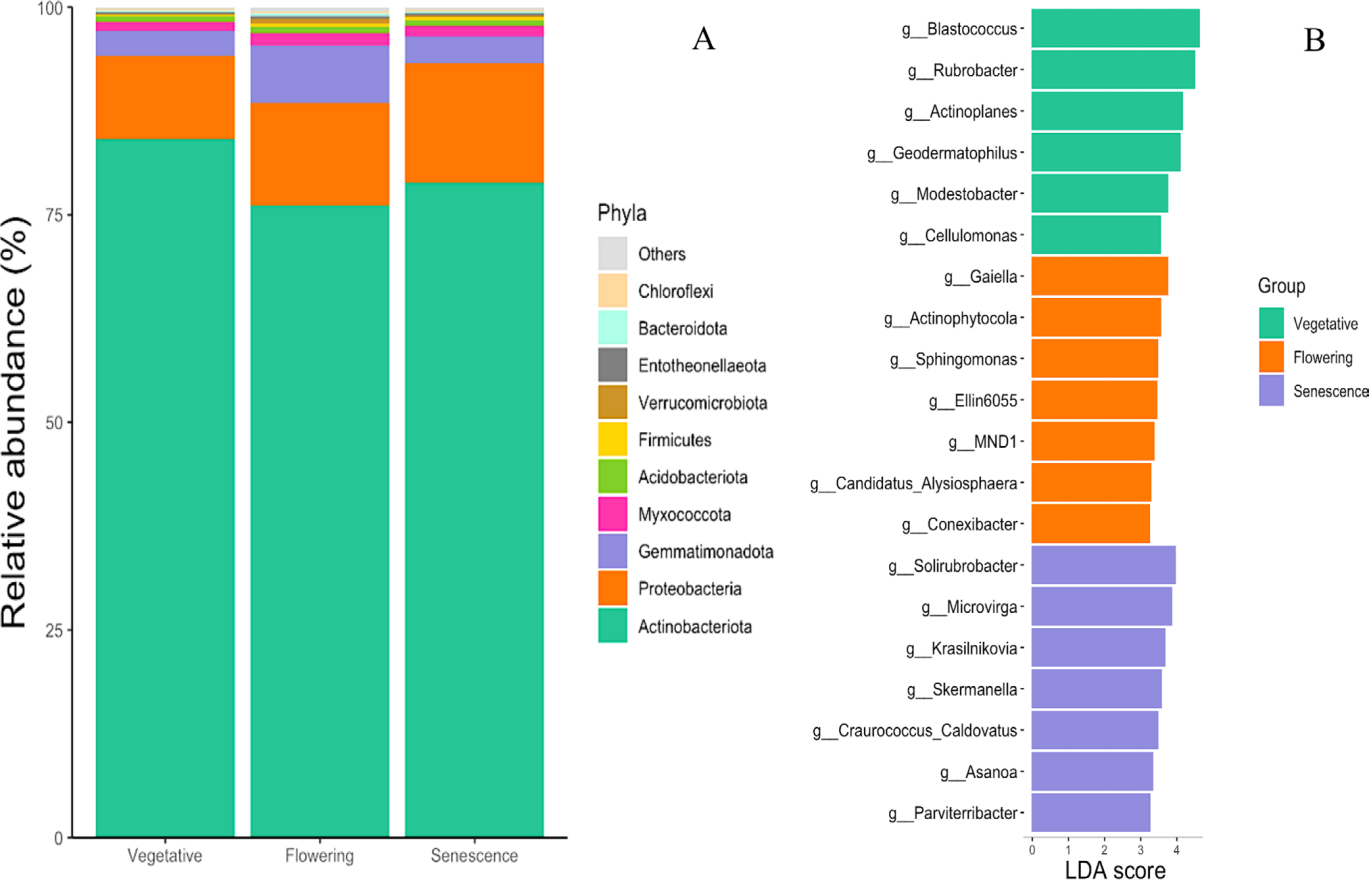
Relative abundances of the most bacterial phyla (A) and genera (B) identified in the rhizosphere of *L. dentata* at different developmental stages.

Soil bacteria were classified into 24 phyla, including Actinobacteria, Proteobacteria, Gemmatimonadota, Myxococcota and Acidobacteria. The Actinobacteria phylum was the most abundant regardless of the development stage of *L. dentata* , with the highest total bacteria OTUs recorded at the vegetative stage (84.11%). However, it decreased significantly (*p* < 0.0001) at flowering (76.09%) and senescence (78.87%) stages (Figure 3A). Proteobacteria phylum abundance increased at senescence (14.35%) compared to the vegetative (10.03%) and flowering (12.35%) stages. Gemmatimonadota phylum was less abundant at the vegetative (2.98%) and senescence (3.24%) than at flowering (6.90%) (Figure 3A). Furthermore, Fibrobacterota and Terrabacteria phyla were only present at the vegetative stage, whereas Bdellovibrionota were only detected at flowering and senescence. The Methylomirabilota and Elusimicrobiota phyla were exclusively identified at senescence.

At the genus level, *Blastococcus*, *Solirubrobacter*, *Rubrobacter* and *Actinoplanes* were the most represented genera in the rhizospheric soil of *L. dentata* , together accounting for almost 50% of the bacterial community, whereas more than 300 genera accounted for the remaining 50% (Figure S2). The abundance of genera varied significantly between the different development stages. Some genera were more abundant at the vegetative stage, such as *Blastococcus*, *Rubrobacter*, *Actinoplanes*, *Geodermatophilus*, *Modestobacter* and Cellulomonas (Figure 3B). Genera like *Gaiella*, *Actinophytocola*, *Sphingomonas* and *Conexibacter* were more abundant at the flowering stage. Meanwhile, the senescence stage was characterised by the abundance of other genera, such as *Solirubrobacter*, *Microvirga*, *Krasilnikovia* and *Skermanella* (Figure 3B).

#### 3.4.2 Fungi

**FIGURE 4:**
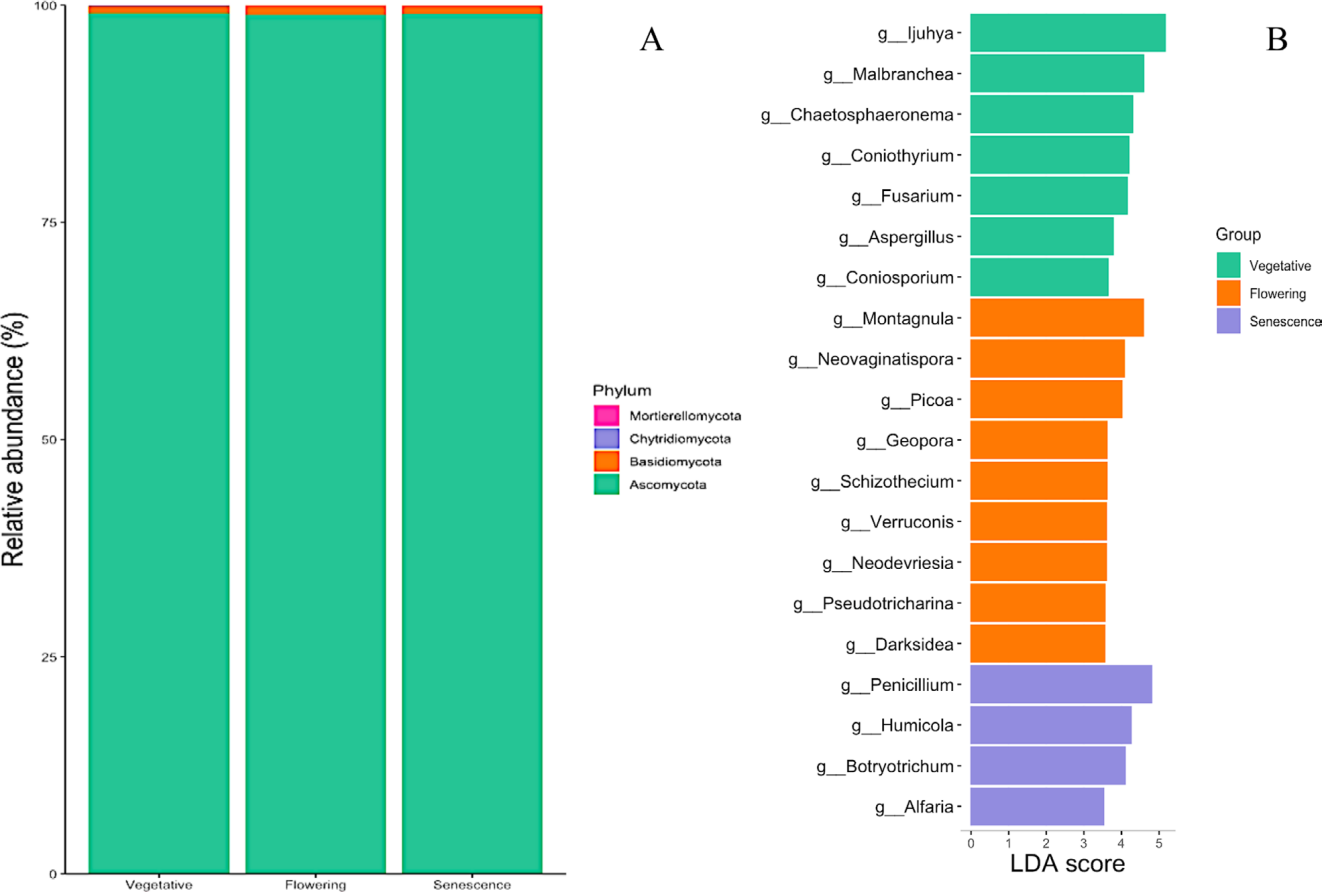
Relative abundance of the main fungal phyla (left) and genera (right) identified in the rhizosphere of *L. dentata* at different developmental stages.

Regarding soil fungal OTUs, three phyla were identified: Ascomycota, Basidiomycota and Chytridiomycota. Irrespective of *L. dentata* 's development stages, Ascomycota was the most represented phylum, accounting for more than 90% of the total fungal OTUs. Other identified phyla displayed significantly lower relative abundances, below 4%, regardless of the development stage (Figure 4A).

No significant differences were observed between the development stages regarding the relative abundance of Ascomycota and Basidiomycota. However, at the genus level, *Penicillium*, *Humicola, Ijuhya, Venturia, Knufia, Alternaria* and *Chaetosphaeronema* were the most represented genera, collectively accounting for almost half of the soil bacterial communities (Figure S4). The second half consisted of the 260 other genera. *Penicillium, Humicola and Alfaria* were 3, 3.5 and 4 times more abundant at senescence, compared to the vegetative stage which was characterised by the abundance of *Ijuhya, Malbranchea, Chaetophaeronema, Coniothryrium, Fusarium, Aspergillus* and *Coniosporium*. In contrast, the flowering stage was characterised by a high abundance of *Montagnula, Neovaginatispora, Picoa, Geopora, Schizothecium* and *Verruconis* among others (Figure 4B).

#### 3.4.3 AMF

**FIGURE 5:**
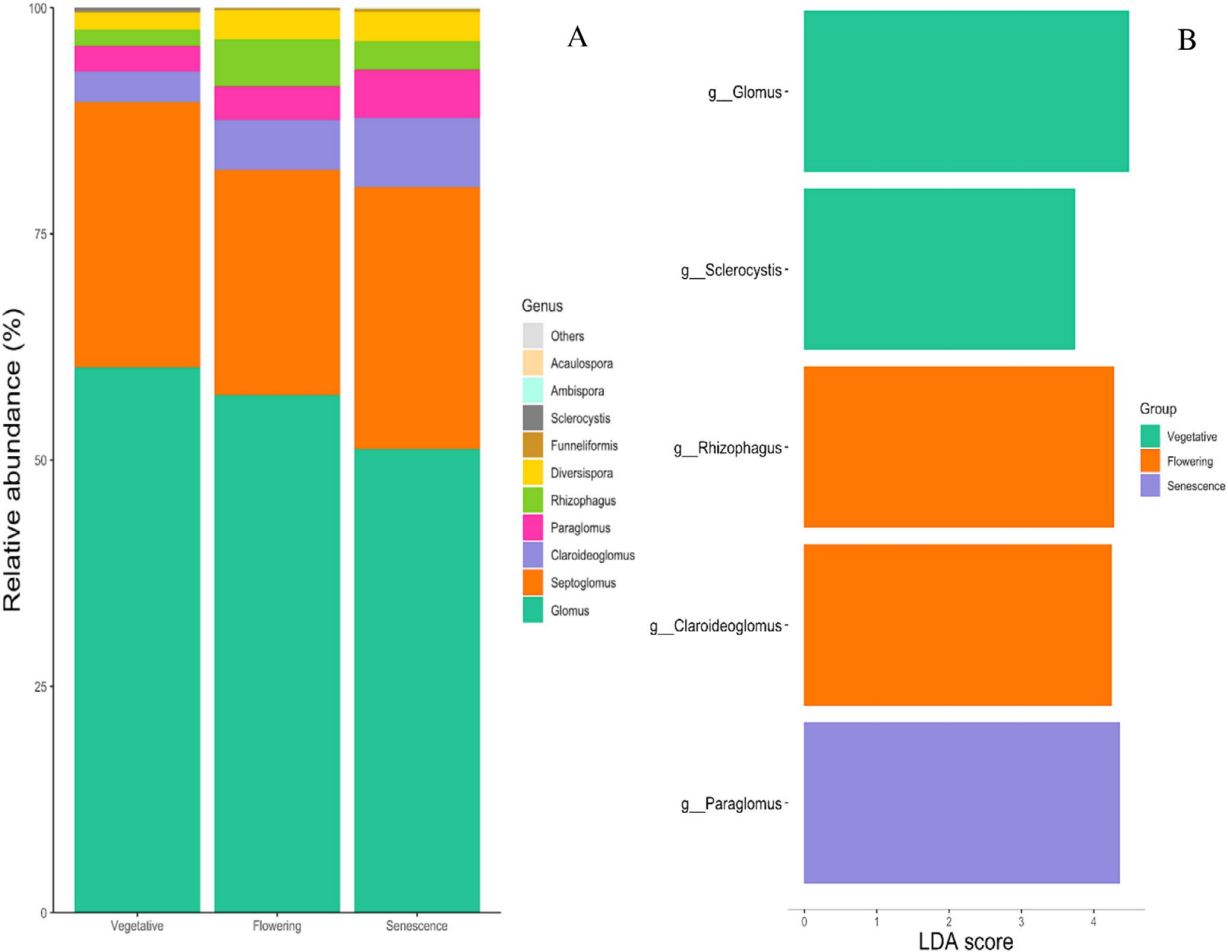
Relative abundances of the main AMF genera identified in the rhizosphere of *L. dentata* at different developmental stages.

Regarding soil AMF OTUs, we focused our analysis on Mucoromycota as the phylum to which mycorrhizal fungi belong. At the genus level, we identified 20 genera, with the most abundant genus being *Glomus*, followed by *Septoglomus, Claroideoglomus, Diversospora, Paraglomus* and *Rhizophagus*. Together, they accounted for almost 95% of the soil AMF communities (Figure 5A). The flowering stage, with 13 genera, was richer than the vegetative (9 genera) and senescence (11 genera) stages. Some genera were identified as specific to a defined development stage. For example, *Glomus* and *Sclerocystis* were specific to the vegetative stage, *Rhizophagus* and *Claroideoglomus* were specific to flowering, and *Paraglomus* to senescence (Figure 5B).

### 3.5 Prediction of OTU Functional Assignments

#### 3.5.1 Prediction of the Ecological Functions of Bacterial Communities

**FIGURE 6:**
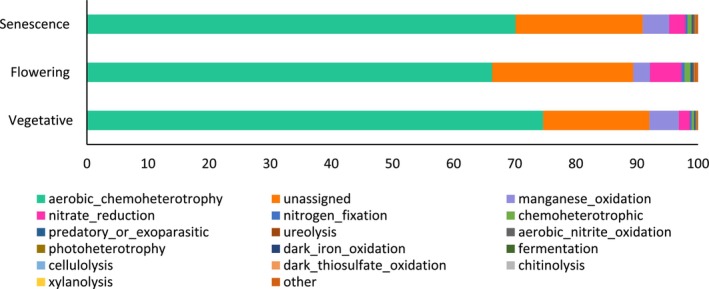
Relative abundances (%) of the major bacterial functional groups identified in the rhizosphere of *L. dentata* at different developmental stages.

Among the 390 bacterial genera identified, at least 75% were successfully assigned to a functional guild, according to FAPROTAX. This functional assignment revealed a total of 19 functional groups. Regardless of the development stages, aerobic chemoheterotrophy was the major group, accounting for up to 74.64% of the total abundance (Figure 6). The abundance of the different functional bacterial groups, particularly the aerobic chemoheterotrophy and nitrogen fixation groups, did not vary between the vegetative and flowering stages. However, the abundance of nitrate reduction and chemoheterotrophic groups increased and the manganese oxidation group decreased during flowering compared to the vegetative stage. On the other hand, the abundance of the aerobic chemoheterotrophy and manganese oxidation groups was higher at senescence than at flowering, while the abundance of the other groups did not significantly change according to the development stage of *L. dentata* .

#### 3.5.2 Prediction of the Ecological Functions of Fungal Communities

**FIGURE 7:**
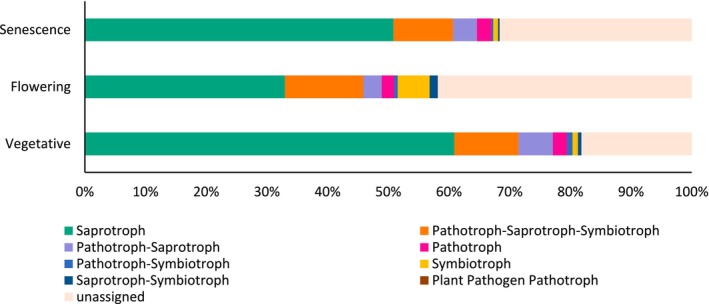
Relative abundances (%) of the major fungal functional groups identified in the rhizosphere of *L. dentata* at different developmental stages.

Among the 268 genera identified, at least 58% were successfully assigned to a functional guild using FUNGUILD, with a ‘probable’ or higher confidence ranking. This functional assignment revealed 7 functional groups, with Saprotroph being the major group regardless of the development stage, accounting for up to 60.86% of the total abundance. The other functional groups, in order of decreasing abundance, were Pathotroph‐Saprotroph‐Symbiotroph, Pathotroph‐Saprotroph, Pathotroph, Pathotroph‐Symbiotroph, Symbiotroph, Saprotroph‐Symbiotroph and Plant Pathogen‐Pathotroph (Figure 7). The abundance of most of these fungal functional groups did not vary significantly among the different development stages.

However, Smybiotroph was the only functional group with significantly increased abundance at flowering compared to the vegetative and senescence stages. Additionally, the abundance of Saprotroph and Pathotroph‐Saprotroph‐Symbiotroph increased at senescence compared to flowering.

### 3.6 Soil Microbial Network Complexity

**FIGURE 8:**
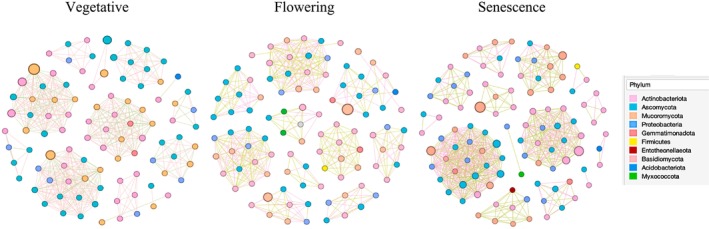
Co‐occurrence network of soil bacterial and fungal communities in the rhizosphere of *L. dentata* at different developmental stages. The nodes are coloured by Phylum. Node size is proportional to relative abundance. Positive links are coloured in light green, negative links are coloured in pink. Table S2 shows the taxonomic identification for each node and its number of interactions.

The impact of developmental stage on soil microbial interaction was assessed by constructing interkingdom networks using Gephi. The analysis of the networks showed the presence of specific phyla, which we defined as keystone taxa present in all networks regardless of the development stage: Actinobacteria, Proteobacteria, Gemmatimonadota, Acidobacteria, Ascomycota and Mucoromycota. These phyla were present in all constructed interaction networks, although with varying abundances across the development stages (Figure 8).

At senescence, the network underwent a significant increase in complexity, with a high number of nodes reaching up to 104 (Table 1). Among these, 56 nodes were bacteria, belonging to the previously mentioned bacterial phyla, in addition to Myxococcota, Firmicutes and Entotheonellaeota. Although 29 nodes were fungi, primarily belonging to Ascomycota, with 20 nodes representing AMF. The network also exhibited a high number of edges, reaching up to 574, with 56% of them being positive and 44% negative (Table 1). The flowering stage featured 99 nodes: 50 were bacterial, sharing keystone taxa with senescence except Entotheonellaeota. Verrucomicrobiota also influenced network interactions. Additionally, 30 nodes were fungi (Ascomycota) and 19 were AMF. The edges in this stage reached up to 418, with 58% being positive and 42% negative. At the vegetative stage, the network was less complex, with 96 nodes, with 40 of these nodes being bacterial, belonging to Actinobacteria, Proteobacteria, Gemmatimonadota and Acidobacteria and 34 nodes were fungi (Ascomycota) and 22 represented AMF. Additionally, 481 edges were noted, with 62% being positive and 38% negative (Table S2).

**TABLE 1:** Number of nodes and edges (B) in co‐occurrence interkingdom network in the rhizosphere of *L. dentata* at different developmental stages.

		Vegetative	Flowering	Senescence
Number of nodes		96	99	104
Number of edges	Number of negative edges	182.78	175.56	252.56
	Number of positive edges	298.22	242.44	321.44
	Number of total edges	481	418	574

## 4 Discussion

*Lavandula dentata* is an aromatic and medicinal Mediterranean shrub that survives and thrives in semi‐arid regions (northwest of Africa, south and east of Spain, Jordan, Ethiopia and the Arabian Peninsula) where it has to withstand the harsh conditions of the natural environment (Nuru et al. 2015). It typically thrives on deep, rocky calcareous soils at elevations below 1550 m (Jaafar 1994). It is known for its tolerance to salinity, as it is highly recommended for cultivation in areas with saline water for irrigation (Paraskevopoulou et al. 2020). These species typically exhibit limited cold tolerance in temperate regions, particularly in northern climates where recurrent frost events are common (Van Oost et al. 2023). In regard to its classification on the IUCN Red List, *L. dentata* is not currently listed as a threatened species, and is not (to date) included in the IUCN's global priority categories. However, the vulnerability assessment approach based on 182 inventoried species indicates that *L. dentata* requires targeted actions for its conservation, ecological restoration, sustainable management, value chain structuring and valorization (Lamrani‐Alaoui and Hassikou 2018). Under these extremely severe conditions, plants can survive with the help of associated soil microbiota. Thus, the present study aims to investigate the influence of the developmental stages (vegetative, flowering and senescence) on microbial communities in the rhizospheric soil of *L. dentata* collected from a Moroccan semi‐arid mountainous region with high 1400 m altitude, 368 mm precipitation, and temperature ranging between −8°C and 33°C.

### 4.1 Senescence Promotes the Microbial Biomass in the Rizosphere of *L. dentata*

**FIGURE 9:**
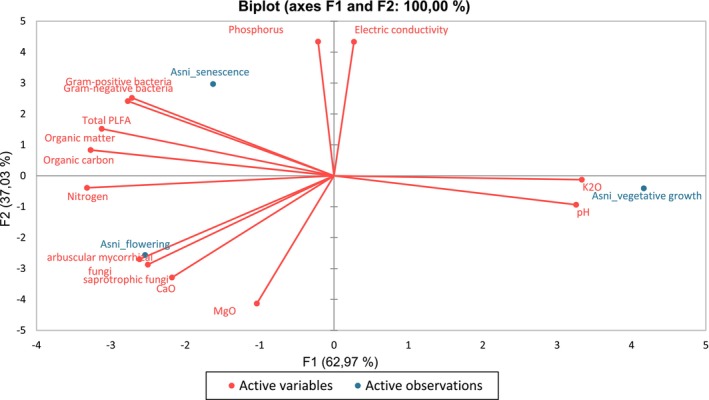
Correlation circles from the principal component statistical analyses (PCA) on the total PLFA, bacterial and fungal biomasses variation according to *L. dentata* developmental stages.

Our findings showed that total microbial biomass was higher at senescence than at the flowering and vegetative stages. These variations of microbial biomass versus developmental stage could be related to the difference in nutrients (P, N, etc.) availability between development stages (Kou et al. 2018). Indeed, a positive correlation was pointed out between available nutrients and total microbial biomass (ACP 1, Figure 9). Nutrient contents were higher at the senescence and flowering stages than at the vegetative stage. Previous studies have shown that the need for nutrients depends on the plant's developmental stage. Indeed, plant nutrient requirements grow during the reproductive stage and dry matter accumulation (Bender et al. 2013; Dufour and Guérin 2005). Moreover, plants release root exudates according to their requirements (Dufour and Guérin 2005). Root exudates, consisting of easily available organic substances, stimulate the growth and activity of soil microbiota, which act as drivers of nutrient transformation, including P solubilisation, nitrogen mineralisation and CO_2_ release (Ma, Tang, et al. 2022; Smain and Senegra 2022).

Moreover, bacterial biomass was more abundant than fungal biomass (saprotrophic fungi and AMF) which is consistent with previous studies (Eisenhauer et al. 2017; Kou et al. 2018), showing that bacteria can metabolise the most of the available OM (soluble and easily decomposable) compared to fungi (Paterson et al. 2007; Moore‐Kucera and Dick 2008; Kou et al. 2018). In addition, Gram‐negative bacteria were found to be more abundant than Gram‐positive bacteria in most of the development stages. This result can be explained by the fact that Gram‐negative bacteria depend more on plant‐derived carbon, such as simple carbon compounds, whereas Gram‐positive bacteria depend on more complex carbon forms (carbonyls) (Kramer and Gleixner 2006; Bird et al. 2011; Fanin et al. 2019). (Wang et al. 2018).

AMF biomass content was higher at flowering, while no significant differences were observed between the vegetative and senescence stages. According to PCA (Figure 9), a positive correlation was noted between AMF biomass and soil nitrogen, which is more available at flowering and senescence soil than in the vegetative stage. Similar results were obtained by Wang et al. (2018), confirming the increase of AMF biomass with the increase of soil nitrogen.

### 4.2 Senescence and Flowering Stages Promote Microbial Diversity and Richness in *L. dentata* Rhizosphere

**FIGURE 10:**
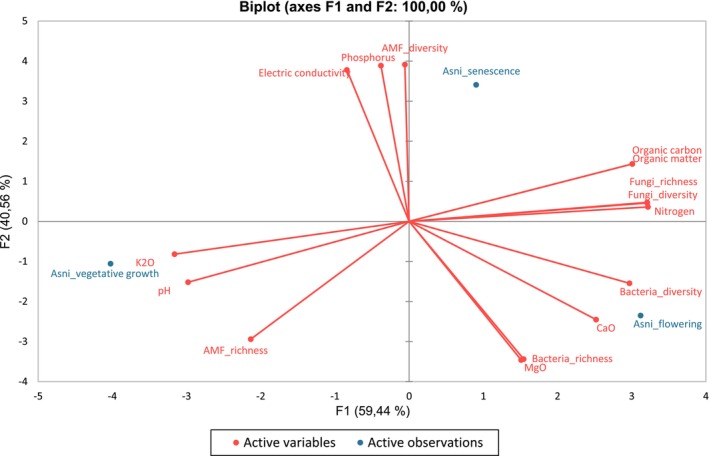
Correlation circles from the principal component statistical analyses (PCA) on bacteria and fungi richness and diversity variations according to *L. dentata* development stages.

The rhizospheric microbial diversity and richness were higher during senescence and flowering than during the vegetative stage. This result might be related to the highest soil OM content at these developmental stages. Plant debris and root exudates, rich in hydrocarbon substances, increase at flowering and senescence (Dufour and Guérin 2005; Ferry et al. 2014), consequently stimulating soil microbiota and contributing to the increase of OM (Ferry et al. 2014). Moreover, these microorganisms release organic substances into the soil mainly polysaccharides, which also increase the rate of OM (Schulz et al. 2013). Indeed, according to the PCA analysis (Figure 10), a high positive correlation was detected between the microbial diversity and richness and the OM content for both bacteria and saprotrophic fungi. These results are in agreement with previous studies that reported the increase of diversity and richness of soil microorganisms by increasing the available organic carbon (Landa et al. 2013; Xiao et al. 2019). However, AMF richness was negatively correlated with soil phosphorus content (Figure 10). These findings indicate that the consistently low phosphorus levels, especially during the vegetative (0.008 g/kg) and flowering (0.007 g/kg) stages, may heighten the plant's reliance on AMF to improve phosphorus uptake efficiency. In a phosphorus‐rich environment, plants use the direct uptake pathway of P by roots, ignoring the AMF pathway which may negatively impact AMF richness (Camenzind et al. 2014; Ma, Chen, et al. 2022).

On the other hand, higher soil pH levels (up to 8.08) were associated with lower richness and diversity of the rhizosphere microbiota (Figure 10). It is known that soil pH is a critical factor for microbial development and a predictor of biogeographical microbial patterns (Xia et al. 2020). The soil pH could also decrease the availability of mineral cations in the soil, which decreases acid‐sensitive microbial communities, resulting in the loss of rare species (Kaspari et al. 2017; Xia et al. 2020). The optimal soil pH for microbial growth should range between 6 and 7.5 (Dewangan et al. 2023).

### 4.3 Increase in Actinobacteria at the Senescence Stage of *L. dentata*

Actinobacteria, Proteobacteria, Gemmatimonadota, Myxococcota and Acidobacteria were the most important phyla in the soil independently of the *L. dentata* developmental stage (Chaparro et al. 2014). However, their abundances varied significantly according to the development stage. Actinobacteria was the dominant phylum at all development stages compared to other phyla. This is the case in the most arid regions and suppressive soils, probably due to the ability of Actinobacteria to form resistant actinospores (Carrasco et al. 2010; Zvyagintsev et al. 2007). It was mainly represented by Geodermatophilaceae, Micromonosporaceae and Rubrobacteriaceae families. The Actinobacteria abundance was highest at the vegetative and senescence stages, and lowest at flowering. This result is consistent with the study of Chaparro et al. (2014), who showed that Actinobacteria abundance increased at seedling and vegetative stages and decreased at bolting and flowering stages in Arabidopsis. Different parameters, including soil physicochemical factors, could explain this variation. For example, the increase in the C/N ratio favours the Actinobacteria abundance (Ren et al. 2022). Indeed, this can also explain the differences observed between the development stages in Lavender, which exhibit high C/N ratio values of 22 at the vegetative stage, 20 at senescence, and 19 at flowering. Actinobacteria grow faster in nutrient‐rich environments compared to the oligotrophic taxa. They are the carbon metabolizers and hence, more abundant in soils with high available carbon content (Fierer et al. 2007; Leff et al. 2015). Moreover, the high abundance of Actinobacteria during senescence may be due to the high OM (73.39 g/kg) and organic carbon (42.67 g/kg) contents.

The high occurrence of the Actinobacteria phylum in most soils worldwide has led to an understanding of its role. Actinobacteria are plant growth promoters and have been shown to improve root proliferation, shoot length and dry biomass accumulation (Rao et al. 2022). Their presence has been correlated with the availability of nutrients and can contribute to nitrogen fixation (Dahal et al. 2017). They can also solubilise potassium and phosphorus via acidification and produce siderophores and polysaccharides (Etesami et al. 2017). Moreover, Actinobacteria produce more than 20,000 secondary metabolites with antifungal and antimicrobial activities that can strengthen plant resistance against plant pathogens (Ibnouf et al. 2022).

### 4.4 Rhizosphere Fungi Are Less Affected by *L. dentata* Developmental Stages Than Bacteria

Ascomycota was the dominant fungal phylum, followed by Basidiomycota, whatever the *L. dentata* development stages. No significant differences were observed in the abundance of Ascomycota and Basidiomycota regarding the development stages of lavender. Ascomycota are the principal decomposers of OM thanks to their various enzymes (alkaline phosphatase, α‐glucosidase, N‐acetylglucosaminidase, etc.) (Eichlerová et al. 2015; Lin et al. 2019). They also produce secondary metabolites to protect their host plants against pathogens (Jia et al. 2020). The predominance of Ascomycota and Basidiomycota has already been reported in different stressful conditions, such as the Actama desert, located in northern Chile, which is the driest region in the world with an average annual rainfall of 36.7 mm (Fuentes et al. 2020). The difference in abundance is more pronounced at the genus level, as observed with *Penicillium*, which is more abundant at senescence than at other stages. *Penicillium* is known for its biocontrol ability against *Fusarium* and *Verticillium* wilt (Jabnoun‐Khiareddine et al. 2010). This increase in abundance at senescence may be related to the occurrence of pathogenic genera such as *Venturia* (Khajuria et al. 2022). Indeed, the senescence stage coincides with summer, where temperature and humidity conditions are more suitable for the occurrence of phytopathogenic genera (Wyka et al. 2018). Our findings imply that physicochemical soil factors might influence the structure of the fungal community.

Regarding AMF variation, the Mucoromycota phylum was more abundant at flowering and senescence stages when plant nutrient needs were higher (Bender et al. 2013; Dufour and Guérin 2005). It is well‐known that AMFs are crucial in improving nutrient uptake, especially in nutrient‐poor soils (Cera et al. 2021). Furthermore, the variation in genera abundance at each development stage could contribute to the functional compatibility of AMF, depending on the benefits expected by the host plant at each stage (Ravnskov and Jakobsen 1995). For example, the vegetative stage was characterised by the abundance of *Glomus*, generally more represented during early growth stages due to its rapid colonisation capacity but slower internal root growth. However, as observed in this study, it was mentioned that the relative abundance of this species decreases with plant age (Kjøller and Rosendahl 2001). The genus *Rhizophagus* was found to be more abundant at flowering, when the plant's phosphorus needs are higher due to its reallocation to reproductive organs (Bucciarelli et al. 2006; Kohout et al. 2015). The *Paraglomus* genus was found to be more abundant at the senescence stage. This result is consistent with a previous study reporting an increased abundance of *Paraglomus* sp. with plant age, which was related to the colonisation speed (Yu et al. 2012). Moreover, the allocation of plant photosynthates to AMF could be an important factor in the dynamics of the AMF community. The most competitive AMF could have an advantage in colonising plant roots, thus influencing the AMF community composition (Verbruggen et al. 2012).

### 4.5 Soil Microbial Interactions Became More Complex at the Senescence Stage

Our results showed that the soil microbial interactions became more complex (regarding the number of nodes and edges) during senescence, followed by flowering, than during the vegetative stage. Various mechanisms can contribute to the expansion and complexity of networks in the rhizosphere. In fact, the senescence stage coincides with summer, when high temperatures and low soil moisture reduce nutrient availability (Mishra et al. 2023). To cope with these stresses, the plant may adopt two complementary strategies: first, by increasing carbon allocation to its root system, as previously reported under high‐temperature and drought conditions (Xia et al. 2023), which could be confirmed by the high level of organic carbon recorded at the senescence stage (42.67 g/kg) compared to the other stages; and second, by enhancing symbiotic interactions with beneficial soil microorganisms, such as mycorrhizal fungi and rhizobia, which improve nutrient uptake and stress resilience in arid environments (Molefe et al. 2023). Moreover, the positive correlation observed between organic carbon and organic matter in our PCA (PCA1 and PCA2; Figures 9 and 10) further supports the idea that senescence is a key phase for carbon‐driven microbial activation in the rhizosphere. The availability of OM during senescence (73.39 g/kg) could also favour the complexity of the interkingdom network at this stage, followed by flowering as the second stage with high OM. Various studies showed that resource and food availability are crucial in structuring networks (Narsing Rao et al. 2022). The abundance of OM, a complex nutrient matrix, requires decomposition and transformation by diverse microbial communities. In particular, aerobic chemoheterotrophic bacteria and saprotrophic fungi, which play key roles in OM degradation and nutrient recycling, were found to be more abundant at the senescence stage compared to other developmental phases. This observation supports our hypothesis and helps explain the increased microbial biomass and activity observed at senescence, when the accumulation of organic substrates likely stimulates the growth of these functional groups. This process makes nutrients accessible to plants and other organisms, leading to both beneficial and detrimental interactions, explaining the results obtained (Suleiman et al. 2019).

Furthermore, interesting interactions are observed between the different kingdoms (bacteria and fungi) regardless of the development stage. Bacteria and fungi displayed closer associations within their respective communities, suggesting a wide range of interactions between species, including metabolite exchange (Woyke et al. 2006; Yang et al. 2022). Generally, compounds released by fungi during the breakdown of recalcitrant OM in the soil, such as water‐soluble sugars and phenolic compounds, can be utilized by bacteria (de Boer et al. 2005). Although fungi contribute to producing some carbon, plant root exudates containing amino acids, sugars and organic acids are considered the primary carbon source for bacteria (Philippot et al. 2013). However, the dominance of bacteria nodes across development stages was highly notable. This situation is likely due to their significantly broader range of physiologies, such as chemoautotrophy and nitrogen fixation, in contrast to fungi and other eukaryotes (Fanin et al. 2019; Schmidt et al. 2014).

Additionally, many positive edges were observed during the vegetative stage, followed by flowering and then senescence. Our results are consistent with previous studies showing a decrease in positive edges between bacteria and fungi over time, indicating an escalating competition between these organisms throughout plant development stages (Xiong et al. 2021). Moreover, the decrease of positive edges from the vegetative to the senescence could stem from abiotic variations (such as niche heterogeneity) within the environment of the studied communities (Götzenberger et al. 2012; Brazeau and Schamp 2019; Ceja‐Navarro et al. 2021). We suggest that the increased proportion of negative correlations at senescence is due to greater soil heterogeneity.

## 5 Conclusion

This study highlights the shifts in rhizospheric microbial communities associated with *L* . *dentata* across its developmental stages in a semi‐arid environment. Our results show that total microbial biomass, as well as microbial diversity and richness, increase significantly at the flowering and senescence stages. While Actinobacteria were particularly abundant during the vegetative and senescence phases, fungal communities appeared less sensitive to plant developmental changes compared to bacterial communities. Additionally, microbial interaction networks became more complex and heterogeneous at senescence, suggesting adaptive microbial responses to shifts in plant physiology and environmental conditions. These findings provide valuable insights for developing sustainable management strategies for *L. dentata* , particularly in arid and semi‐arid regions. Optimising the timing and composition of organic amendments or microbial inoculants in accordance with plant phenology could support beneficial microbial communities involved in nutrient cycling, stress tolerance and disease suppression. Such microbiome‐informed approaches offer the potential to reduce reliance on synthetic inputs and enhance both the ecological resilience and productivity of this endangered aromatic species. Finally, understanding the microbial patterns linked to key developmental stages, especially flowering and senescence, opens new perspectives for the use of targeted microbial consortia to improve resource efficiency and the sustainable cultivation of *L. dentata* .

## Author Contributions

Conceptualization, O.A, A.L.‐H.S., A.Q. and H.B.; methodology and validation, O.A, J.L., H.B., N.F, F.L, A.Q. and A.L.‐H.S.; Software, O.A.; writing – original draft preparation, O.A.; writing – review and editing, O.A, A.L.‐H.S, A.Q. and H.B.; supervision, H.B., A.L.‐H.S. and A.Q. All authors have read and agreed to this version of the manuscript.

## Funding

This work was supported by project PHC‐TOUBKAL/21/115‐Campus France (45884PG), VPMA4 Moroccan project, Alibiotech, Bio‐TEAM (ANR‐25‐PE and BiHauts Eco de France projects).

## Consent

The authors have nothing to report.

## Conflicts of Interest

The authors declare no conflicts of interest.

## Supporting information


**Data S1:** emi470318‐sup‐0001‐Supinfo.docx.

## Data Availability

The data that support the findings of this study are available on request from the corresponding author. The data are not publicly available due to privacy or ethical restrictions.
