# Supplementary material for: Comparative Analysis of Lavandula Dentata Rhizosphere Microbiota Across Different Developmental Stages in a Semi‐Arid Area
Source: Environ Microbiol Rep. 2026 Mar 17;18(2):e70318. doi: 10.1111/1758-2229.70318 (PMC13053143; doi:10.1111/1758-2229.70318)
Supplement: Supplementary file 1 — Data S1: emi470318‐sup‐0001‐Supinfo.docx. [file EMI4-18-e70318-s001.docx]

**Supplement data**

Table 1S: Characteristics of the rhizospheric soil of L. dentata, at different development stages: vegetative, flowering, and senescence..

|  | **Development**  **Stage** | **pH watter** | **pH KCl** | **Organic matter**  **(g/kg)** | **Organic Carbon**  **(g/kg)** | **Nitrogen (g/kg)** | **C/N** | **Total limestone (g/kg)** | **CEC (méq/Kg)** | **Calcium (g/kg)** | **Potassium**  **(g/kg)** | **Magnesium**  **(g/kg)** | **Phosphorus**  **(g/kg)** |
| --- | --- | --- | --- | --- | --- | --- | --- | --- | --- | --- | --- | --- | --- |
| ***L. dentata*** | Vegetative | 8.08 | 7.60 | 65.76 | 38.23 | 1.72 | 22 | 214 | 152.39 | 11.38 | 0.375 | 0.504 | 0.008 |
|  | Flowering | 7.99 | 7.57 | 72.82 | 42.34 | 2.21 | 19 | 223 | 197.68 | 13.25 | 0.328 | 0.582 | 0.007 |
|  | Senescence | 7.98 | 7.55 | 73.39 | 42.67 | 2.1 | 20 | 280 | 178.76 | 11.51 | 0.333 | 0.465 | 0.01 |


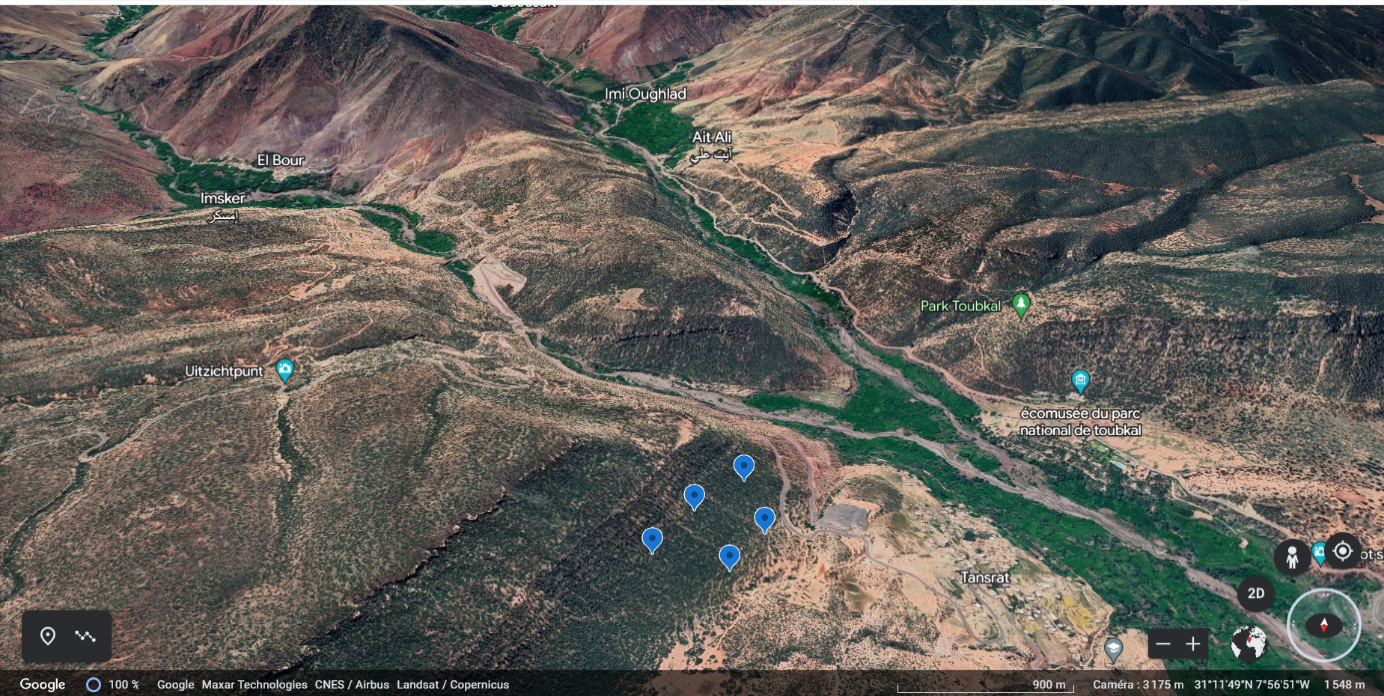


Figure 1S: Sampling sites for soil samples in the ASNI regions.


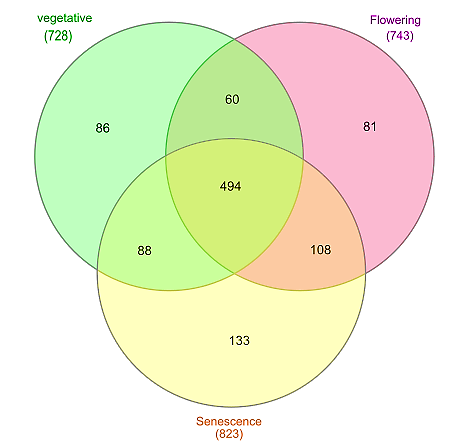


Bacterial


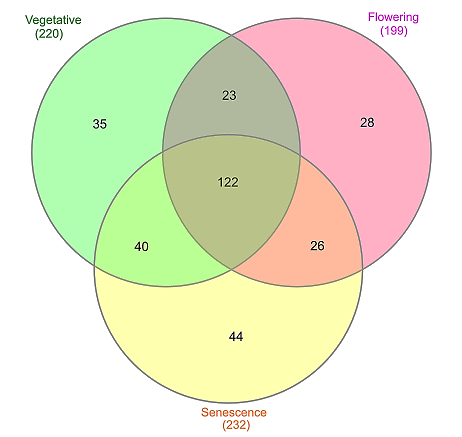


Fungal


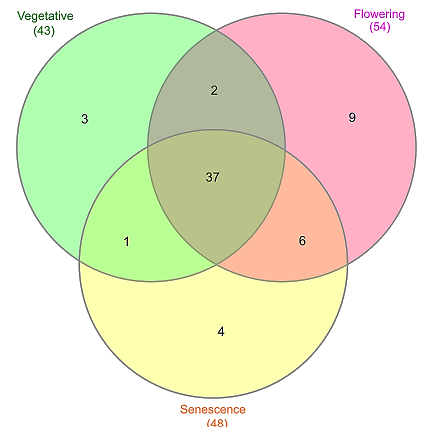


AMF

Figure 2S: Venn diagrams showing the overlap of the bacterial. fungal. and AMF communities in Asni at different L. dentata’s development stages.


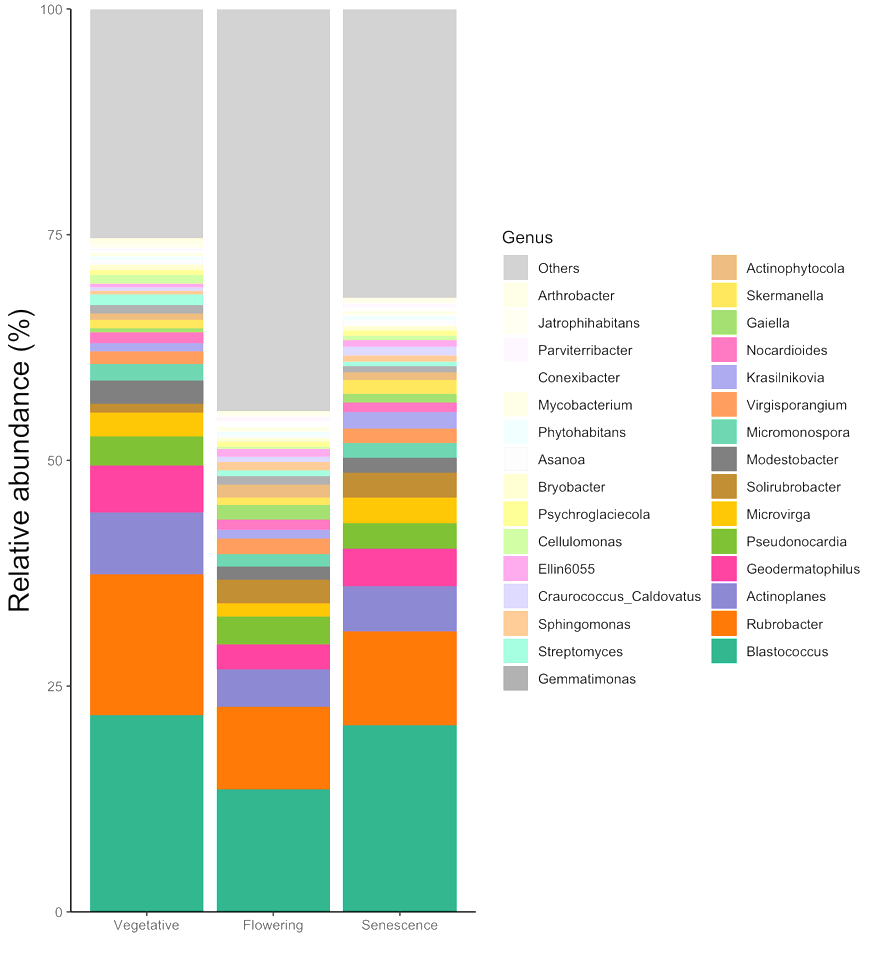

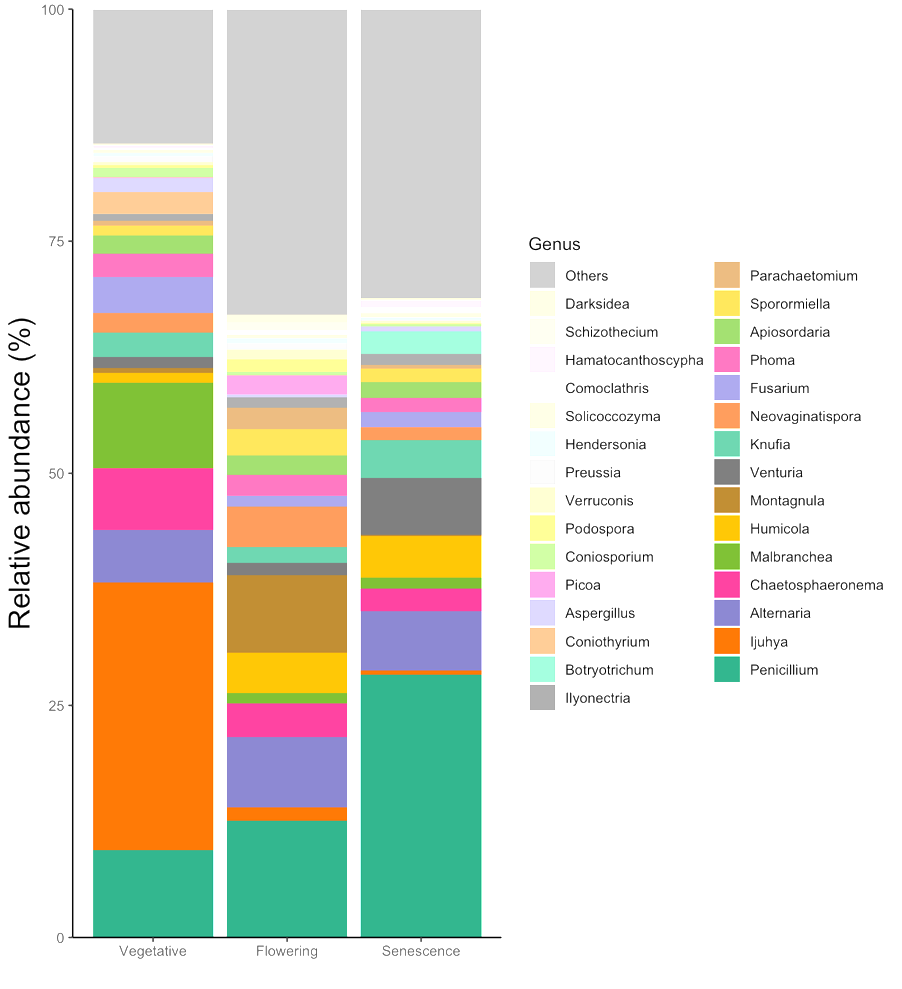


Figure 3S: Relative abundances of the most bacterial phyla and genera identified in Asni soil at the different L. dentata’s development stages

Figure 4S: Relative abundances of the most fungal genera identified in Asni soil at the different L. dentata’s development stages.

Table 2S: Taxonomic identification for each node and its number of interactions.

| Vegetative | | | | |
| --- | --- | --- | --- | --- |
| Id | Taxa | Relative abundance | Number of interactions | |
|  |  |  | Source | Destination |
| 1 | BacteriaAcidobacteriotaAcidobacteriaeBryobacteralesBryobacteraceaeBryobacter | 0.14442231 | 1 | 0 |
| 2 | BacteriaActinobacteriotaAcidimicrobiiaIMCC26256unknown_familyunknown_genus | 0.17679283 | 14 | 0 |
| 3 | BacteriaActinobacteriotaAcidimicrobiiaunknown_orderunknown_familyunknown_genus | 0.1992032 | 5 | 0 |
| 4 | BacteriaActinobacteriotaActinobacteriaCorynebacterialesMycobacteriaceaeMycobacterium | 0.16185258 | 1 | 0 |
| 5 | BacteriaActinobacteriotaActinobacteriaFrankialesFrankiaceaeJatrophihabitans | 0.14691235 | 16 | 0 |
| 6 | BacteriaActinobacteriotaActinobacteriaFrankialesGeodermatophilaceaeBlastococcus | 7.1713147 | 15 | 0 |
| 7 | BacteriaActinobacteriotaActinobacteriaFrankialesGeodermatophilaceaeGeodermatophilus | 1.5961156 | 14 | 1 |
| 8 | BacteriaActinobacteriotaActinobacteriaFrankialesGeodermatophilaceaeModestobacter | 0.80926293 | 13 | 2 |
| 9 | BacteriaActinobacteriotaActinobacteriaFrankialesunknown_familyunknown_genus | 0.5278884 | 4 | 1 |
| 10 | BacteriaActinobacteriotaActinobacteriaKineosporialesKineosporiaceaeunknown_genus | 0.10458168 | 4 | 0 |
| 11 | BacteriaActinobacteriotaActinobacteriaMicrococcalesCellulomonadaceaeCellulomonas | 0.31623507 | 15 | 1 |
| 12 | BacteriaActinobacteriotaActinobacteriaMicrococcalesMicrococcaceaeArthrobacter | 0.14691235 | 3 | 2 |
| 13 | BacteriaActinobacteriotaActinobacteriaMicromonosporalesMicromonosporaceaeActinoplanes | 2.0766933 | 13 | 1 |
| 14 | BacteriaActinobacteriotaActinobacteriaMicromonosporalesMicromonosporaceaeAsanoa | 0.109561756 | 12 | 2 |
| 15 | BacteriaActinobacteriotaActinobacteriaMicromonosporalesMicromonosporaceaeDactylosporangium | 0.114541836 | 5 | 0 |
| 16 | BacteriaActinobacteriotaActinobacteriaMicromonosporalesMicromonosporaceaeKrasilnikovia | 0.25398406 | 4 | 1 |
| 17 | BacteriaActinobacteriotaActinobacteriaMicromonosporalesMicromonosporaceaeLuedemannella | 0.10707171 | 11 | 3 |
| 18 | BacteriaActinobacteriotaActinobacteriaMicromonosporalesMicromonosporaceaeMicromonospora | 0.5428287 | 3 | 2 |
| 19 | BacteriaActinobacteriotaActinobacteriaMicromonosporalesMicromonosporaceaeVirgisporangium | 0.44322708 | 4 | 0 |
| 20 | BacteriaActinobacteriotaActinobacteriaPropionibacterialesNocardioidaceaeMarmoricola | 0.16185258 | 2 | 3 |
| 21 | BacteriaActinobacteriotaActinobacteriaPropionibacterialesNocardioidaceaeNocardioides | 0.33615538 | 0 | 1 |
| 22 | BacteriaActinobacteriotaActinobacteriaPseudonocardialesPseudonocardiaceaeLechevalieria | 0.10209163 | 10 | 4 |
| 23 | BacteriaActinobacteriotaActinobacteriaPseudonocardialesPseudonocardiaceaePseudonocardia | 1.0458168 | 9 | 5 |
| 24 | BacteriaActinobacteriotaActinobacteriaStreptomycetalesStreptomycetaceaeStreptomyces | 0.40836653 | 12 | 3 |
| 25 | BacteriaActinobacteriotaMB_A2_108unknown_orderunknown_familyunknown_genus | 0.35358566 | 1 | 4 |
| 26 | BacteriaActinobacteriotaRubrobacteriaRubrobacteralesRubrobacteriaceaeRubrobacter | 5.4606576 | 11 | 4 |
| 27 | BacteriaActinobacteriotaThermoleophiliaGaiellalesGaiellaceaeGaiella | 0.13446215 | 2 | 0 |
| 28 | BacteriaActinobacteriotaThermoleophiliaGaiellalesunknown_familyunknown_genus | 0.42330676 | 14 | 2 |
| 29 | BacteriaActinobacteriotaThermoleophiliaSolirubrobacterales67_14unknown_genus | 1.0831673 | 8 | 6 |
| 30 | BacteriaActinobacteriotaThermoleophiliaSolirubrobacteralesSolirubrobacteraceaeSolirubrobacter | 0.40338644 | 10 | 5 |
| 31 | BacteriaGemmatimonadotaGemmatimonadetesGemmatimonadalesGemmatimonadaceaeGemmatimonas | 0.41583666 | 7 | 7 |
| 32 | BacteriaGemmatimonadotaGemmatimonadetesGemmatimonadalesGemmatimonadaceaeunknown_genus | 0.52539843 | 6 | 8 |
| 33 | BacteriaProteobacteriaAlphaproteobacteriaAcetobacteralesAcetobacteraceaeCraurococcus_Caldovatus | 0.14193226 | 2 | 3 |
| 34 | BacteriaProteobacteriaAlphaproteobacteriaAzospirillalesAzospirillaceaeSkermanella | 0.2813745 | 13 | 3 |
| 35 | BacteriaProteobacteriaAlphaproteobacteriaRhizobialesBeijerinckiaceaeMicrovirga | 0.90886456 | 12 | 4 |
| 36 | BacteriaProteobacteriaAlphaproteobacteriaRhizobialesBeijerinckiaceaePsychroglaciecola | 0.17430279 | 7 | 0 |
| 37 | BacteriaProteobacteriaAlphaproteobacteriaRhizobialesBeijerinckiaceaeunknown_genus | 0.24651395 | 0 | 5 |
| 38 | BacteriaProteobacteriaAlphaproteobacteriaRhizobialesXanthobacteraceaeunknown_genus | 0.10707171 | 3 | 1 |
| 39 | BacteriaProteobacteriaAlphaproteobacteriaSphingomonadalesSphingomonadaceaeEllin6055 | 0.11205179 | 3 | 1 |
| 40 | BacteriaProteobacteriaAlphaproteobacteriaSphingomonadalesSphingomonadaceaeSphingomonas | 0.119521916 | 6 | 1 |
| 41 | EukaryotaMucoromycotaGlomeromycetesDiversisporalesClaroideoglomeraceaeClaroideoglomusGlomeromycotina_sp_ | 0.13446215 | 5 | 9 |
| 42 | EukaryotaMucoromycotaGlomeromycetesDiversisporalesDiversisporaceaeDiversisporaDiversispora_sp_ | 0.38097608 | 9 | 6 |
| 43 | EukaryotaMucoromycotaGlomeromycetesDiversisporalesDiversisporaceaeDiversisporaDiversispora_spurca | 0.27639443 | 11 | 5 |
| 44 | EukaryotaMucoromycotaGlomeromycetesGlomeralesClaroideoglomeraceaeClaroideoglomusClaroideoglomus_etunicatum | 0.6523904 | 0 | 1 |
| 45 | EukaryotaMucoromycotaGlomeromycetesGlomeralesClaroideoglomeraceaeClaroideoglomusClaroideoglomus_lamellosum | 0.19671315 | 4 | 10 |
| 46 | EukaryotaMucoromycotaGlomeromycetesGlomeralesGlomeraceaeGlomusGlomeraceae_sp_ | 0.39591634 | 3 | 11 |
| 47 | EukaryotaMucoromycotaGlomeromycetesGlomeralesGlomeraceaeGlomusGlomeromycotina_sp_ | 0.3261952 | 8 | 7 |
| 48 | EukaryotaMucoromycotaGlomeromycetesGlomeralesGlomeraceaeGlomusGlomus_mycorrhizal_symbiont_of_Marchantia_foliacea | 1.5039841 | 5 | 2 |
| 49 | EukaryotaMucoromycotaGlomeromycetesGlomeralesGlomeraceaeGlomusmetagenome | 1.5512948 | 10 | 6 |
| 50 | EukaryotaMucoromycotaGlomeromycetesGlomeralesGlomeraceaeGlomusunknown_species | 13.690239 | 7 | 8 |
| 51 | EukaryotaMucoromycotaGlomeromycetesGlomeralesGlomeraceaeRhizophagusGlomeromycotina_sp_ | 0.55278885 | 2 | 12 |
| 52 | EukaryotaMucoromycotaGlomeromycetesGlomeralesGlomeraceaeSeptoglomusGlomeromycotina_sp_ | 9.37998 | 9 | 7 |
| 53 | EukaryotaMucoromycotaGlomeromycetesParaglomeralesParaglomeraceaeParaglomusParaglomus_occultum | 0.14193226 | 4 | 3 |
| 54 | EukaryotaMucoromycotaGlomeromycetesunknown_orderGlomeraceaeGlomusGlomeraceae_sp_ | 0.14442231 | 6 | 9 |
| 55 | EukaryotaMucoromycotaGlomeromycetesunknown_orderGlomeraceaeGlomusGlomeromycotina_sp_ | 1.379482 | 1 | 13 |
| 56 | EukaryotaMucoromycotaGlomeromycetesunknown_orderGlomeraceaeGlomusunknown_species | 2.0866535 | 5 | 10 |
| 57 | EukaryotaMucoromycotaunknown_classMortierellalesMortierellaceaeMortierellametagenome | 0.5229084 | 0 | 14 |
| 58 | EukaryotaMucoromycotaunknown_classMortierellalesMortierellaceaeMortierellaMortierella_alpina | 5.420817 | 1 | 1 |
| 59 | EukaryotaMucoromycotaunknown_classMortierellalesMortierellaceaeMortierellaunknown_species | 0.53037846 | 3 | 4 |
| 60 | EukaryotaMucoromycotaunknown_classMortierellalesParaglomeraceaeParaglomusPhaseoleae_environmental_sample | 0.33615538 | 8 | 0 |
| 61 | EukaryotaMucoromycotaunknown_classMortierellalesParaglomeraceaeParaglomusunknown_species | 0.1942231 | 1 | 0 |
| 62 | EukaryotaMucoromycotaunknown_classMortierellalesunknown_familyModicellaModicella_malleola | 0.2440239 | 2 | 2 |
| 63 | FungiAscomycotaDothideomycetesPleosporalesConiothyriaceaeConiothyriumConiothyrium_dolichi | 0.4307769 | 7 | 1 |
| 64 | FungiAscomycotaDothideomycetesPleosporalesDidymellaceaedidymellaMulti_affiliation | 0.119521916 | 8 | 8 |
| 65 | FungiAscomycotaDothideomycetesPleosporalesDidymosphaeriaceaeMontagnulaMulti_affiliation | 0.14691235 | 1 | 4 |
| 66 | FungiAscomycotaDothideomycetesPleosporalesLophiostomataceaeNeovaginatisporaPleosporales_sp | 0.49551794 | 6 | 2 |
| 67 | FungiAscomycotaDothideomycetesPleosporalesLophiostomataceaeSigarisporaSigarispora_scrophulariae | 0.10209163 | 2 | 2 |
| 68 | FungiAscomycotaDothideomycetesPleosporalesPhaeosphaeriaceaeChaetosphaeronemaChaetosphaeronema_sp | 1.688247 | 4 | 11 |
| 69 | FungiAscomycotaDothideomycetesPleosporalesPhaeosphaeriaceaeParaphomaParaphoma_chlamydocopiosa | 0.14442231 | 5 | 3 |
| 70 | FungiAscomycotaDothideomycetesPleosporalesPleosporaceaeAlternariaAlternaria_chlamydosporigena | 1.3570718 | 7 | 9 |
| 71 | FungiAscomycotaDothideomycetesPleosporalesPleosporaceaeAlternariaAlternaria_subcucurbitae | 0.1319721 | 0 | 1 |
| 72 | FungiAscomycotaDothideomycetesPleosporalesPleosporaceaeunidentifiedPleosporaceae_sp | 0.38346612 | 4 | 4 |
| 73 | FungiAscomycotaDothideomycetesPleosporalesPleosporales_fam_Incertae_sedisPteridiosporaPteridiospora_sp | 0.12201195 | 2 | 5 |
| 74 | FungiAscomycotaDothideomycetesPleosporalesSporormiaceaeSporormiellaSporormiella_intermedia | 0.12201195 | 6 | 10 |
| 75 | FungiAscomycotaDothideomycetesPleosporalesSporormiaceaeSporormiellaSporormiella_similis | 0.20169322 | 1 | 6 |
| 76 | FungiAscomycotaEurotiomycetesChaetothyrialesHerpotrichiellaceaeConiosporiumConiosporium_apollinis | 0.27390438 | 5 | 11 |
| 77 | FungiAscomycotaEurotiomycetesChaetothyrialesTrichomeriaceaeKnufiaKnufia_perforans | 0.50298804 | 3 | 12 |
| 78 | FungiAscomycotaEurotiomycetesChaetothyrialesTrichomeriaceaeKnufiaKnufia_sp | 0.12948208 | 1 | 3 |
| 79 | FungiAscomycotaEurotiomycetesEurotialesAspergillaceaeAspergillusAspergillus_fischeri | 0.2639442 | 4 | 12 |
| 80 | FungiAscomycotaEurotiomycetesEurotialesAspergillaceaePenicilliumPenicillium_novae_zeelandiae | 0.2315737 | 0 | 5 |
| 81 | FungiAscomycotaEurotiomycetesOnygenalesOnygenales_fam_Incertae_sedisMalbrancheaMalbranchea_cinnamomea | 2.8212152 | 3 | 5 |
| 82 | FungiAscomycotaEurotiomycetesOnygenalesunidentifiedunidentifiedOnygenales_sp | 0.23406374 | 2 | 6 |
| 83 | FungiAscomycotaLeotiomycetesHelotialesHamatocanthoscyphaceaeHamatocanthoscyphaMulti_affiliation | 0.119521916 | 3 | 13 |
| 84 | FungiAscomycotaLeotiomycetesHelotialesunidentifiedunidentifiedHelotiales_sp | 0.114541836 | 0 | 7 |
| 85 | FungiAscomycotaMulti_affiliationMulti_affiliationDidymellaceaePhomaMulti_affiliation | 0.5552789 | 1 | 7 |
| 86 | FungiAscomycotaSordariomycetesConiochaetalesConiochaetaceaeConiochaetaConiochaeta_sp | 0.11205179 | 0 | 4 |
| 87 | FungiAscomycotaSordariomycetesGlomerellalesGlomerellaceaeColletotrichumColletotrichum_gloeosporioides | 0.10707171 | 2 | 14 |
| 88 | FungiAscomycotaSordariomycetesHypocrealesBionectriaceaeIjuhyaHypocreales_sp | 7.2833667 | 0 | 2 |
| 89 | FungiAscomycotaSordariomycetesHypocrealesCordycipitaceaeLecanicilliumLecanicillium_sp | 0.12948208 | 1 | 15 |
| 90 | FungiAscomycotaSordariomycetesHypocrealesNectriaceaeFusariumFusarium_sp | 1.0532869 | 0 | 16 |
| 91 | FungiAscomycotaSordariomycetesHypocrealesNectriaceaeIlyonectriaIlyonectria_macrodidyma | 0.15936255 | 0 | 8 |
| 92 | FungiAscomycotaSordariomycetesSordarialesChaetomiaceaeChaetomiumChaetomium_sp | 0.15687251 | 1 | 3 |
| 93 | FungiAscomycotaSordariomycetesSordarialesLasiosphaeriaceaeApiosordariaSordariales_sp | 0.21165338 | 0 | 4 |
| 94 | FungiAscomycotaSordariomycetesunidentifiedChaetomiaceaeParachaetomiumSordariomycetes_sp | 0.13446215 | 2 | 13 |
| 95 | FungiAscomycotaunidentifiedunidentifiedAspergillaceaePenicilliumFungi_sp | 2.2609563 | 1 | 14 |
| 96 | FungiAscomycotaunidentifiedunidentifiedChaetomiaceaeHumicolaAscomycota_sp | 0.25896415 | 0 | 15 |

| Flowering | | | | |
| --- | --- | --- | --- | --- |
| Id | Taxa | Relative abundance | Number of interactions | |
|  |  |  | Source | Destination |
| 1 | BacteriaAcidobacteriotaAcidobacteriaeBryobacteralesBryobacteraceaeBryobacter | 0.17452008 | 4 | 0 |
| 2 | BacteriaActinobacteriotaAcidimicrobiiaIMCC26256unknown_familyunknown_genus | 0.26905176 | 13 | 0 |
| 3 | BacteriaActinobacteriotaAcidimicrobiiaMicrotrichalesIamiaceaeIamia | 0.1145288 | 1 | 0 |
| 4 | BacteriaActinobacteriotaAcidimicrobiiaMicrotrichalesIlumatobacteraceaeunknown_genus | 0.11816463 | 6 | 0 |
| 5 | BacteriaActinobacteriotaAcidimicrobiiaMicrotrichalesunknown_familyunknown_genus | 0.3763089 | 9 | 0 |
| 6 | BacteriaActinobacteriotaAcidimicrobiiaunknown_orderunknown_familyunknown_genus | 0.30722803 | 12 | 1 |
| 7 | BacteriaActinobacteriotaActinobacteria0319_7L14unknown_familyunknown_genus | 0.42175683 | 14 | 0 |
| 8 | BacteriaActinobacteriotaActinobacteriaCorynebacterialesMycobacteriaceaeMycobacterium | 0.17270215 | 8 | 0 |
| 9 | BacteriaActinobacteriotaActinobacteriaFrankialesFrankiaceaeJatrophihabitans | 0.11271088 | 7 | 0 |
| 10 | BacteriaActinobacteriotaActinobacteriaFrankialesGeodermatophilaceaeBlastococcus | 4.18121 | 6 | 0 |
| 11 | BacteriaActinobacteriotaActinobacteriaFrankialesGeodermatophilaceaeGeodermatophilus | 0.81079113 | 8 | 1 |
| 12 | BacteriaActinobacteriotaActinobacteriaFrankialesNakamurellaceaeNakamurella | 0.10180338 | 7 | 0 |
| 13 | BacteriaActinobacteriotaActinobacteriaFrankialesunknown_familyunknown_genus | 0.30541012 | 13 | 1 |
| 14 | BacteriaActinobacteriotaActinobacteriaMicrococcalesMicrococcaceaeArthrobacter | 0.11998255 | 11 | 2 |
| 15 | BacteriaActinobacteriotaActinobacteriaMicromonosporalesMicromonosporaceaeActinoplanes | 1.2089151 | 4 | 0 |
| 16 | BacteriaActinobacteriotaActinobacteriaMicromonosporalesMicromonosporaceaeKrasilnikovia | 0.28541303 | 12 | 2 |
| 17 | BacteriaActinobacteriotaActinobacteriaMicromonosporalesMicromonosporaceaeMicromonospora | 0.46720478 | 11 | 3 |
| 18 | BacteriaActinobacteriotaActinobacteriaMicromonosporalesMicromonosporaceaePhytohabitans | 0.1454334 | 6 | 1 |
| 19 | BacteriaActinobacteriotaActinobacteriaMicromonosporalesMicromonosporaceaeunknown_genus | 0.1781559 | 3 | 1 |
| 20 | BacteriaActinobacteriotaActinobacteriaMicromonosporalesMicromonosporaceaeVirgisporangium | 0.54901105 | 10 | 4 |
| 21 | BacteriaActinobacteriotaActinobacteriaPropionibacterialesNocardioidaceaeKribbella | 0.12725422 | 5 | 1 |
| 22 | BacteriaActinobacteriotaActinobacteriaPropionibacterialesNocardioidaceaeMarmoricola | 0.13452588 | 6 | 1 |
| 23 | BacteriaActinobacteriotaActinobacteriaPropionibacterialesNocardioidaceaeNocardioides | 0.43084642 | 7 | 2 |
| 24 | BacteriaActinobacteriotaActinobacteriaPseudonocardialesPseudonocardiaceaeActinophytocola | 0.39448807 | 3 | 1 |
| 25 | BacteriaActinobacteriotaActinobacteriaPseudonocardialesPseudonocardiaceaePseudonocardia | 0.98894703 | 10 | 3 |
| 26 | BacteriaActinobacteriotaActinobacteriaStreptomycetalesStreptomycetaceaeStreptomyces | 0.25450844 | 1 | 0 |
| 27 | BacteriaActinobacteriotaActinobacteriaStreptosporangialesThermomonosporaceaeActinomadura | 0.1563409 | 6 | 3 |
| 28 | BacteriaActinobacteriotaMB_A2_108unknown_orderunknown_familyunknown_genus | 0.55810064 | 5 | 2 |
| 29 | BacteriaActinobacteriotaRubrobacteriaRubrobacteralesRubrobacteriaceaeRubrobacter | 2.597804 | 9 | 5 |
| 30 | BacteriaActinobacteriotaThermoleophiliaGaiellalesGaiellaceaeGaiella | 0.5071989 | 0 | 1 |
| 31 | BacteriaActinobacteriotaThermoleophiliaGaiellalesunknown_familyunknown_genus | 1.1252909 | 5 | 1 |
| 32 | BacteriaActinobacteriotaThermoleophiliaSolirubrobacterales67_14unknown_genus | 2.0978768 | 4 | 3 |
| 33 | BacteriaActinobacteriotaThermoleophiliaSolirubrobacteralesSolirubrobacteraceaeConexibacter | 0.19088133 | 5 | 4 |
| 34 | BacteriaActinobacteriotaThermoleophiliaSolirubrobacteralesSolirubrobacteraceaeParviterribacter | 0.15815881 | 9 | 4 |
| 35 | BacteriaActinobacteriotaThermoleophiliaSolirubrobacteralesSolirubrobacteraceaeSolirubrobacter | 0.83987784 | 14 | 5 |
| 36 | BacteriaFirmicutesBacilliBacillalesBacillaceaeBacillus | 0.10180338 | 3 | 6 |
| 37 | BacteriaGemmatimonadotaGemmatimonadetesGemmatimonadalesGemmatimonadaceaeGemmatimonas | 6 | 5 | 0 |
| 38 | BacteriaGemmatimonadotaGemmatimonadetesGemmatimonadalesGemmatimonadaceaeunknown_genus | 1.7524724 | 2 | 7 |
| 39 | BacteriaMyxococcotabacteriap25unknown_orderunknown_familyunknown_genus | 0.11271088 | 4 | 2 |
| 40 | BacteriaMyxococcotaPolyangiaHaliangialesHaliangiaceaeHaliangium | 0.1145288 | 3 | 3 |
| 41 | BacteriaProteobacteriaAlphaproteobacteriaAcetobacteralesAcetobacteraceaeCraurococcus_Caldovatus | 0.16543049 | 8 | 6 |
| 42 | BacteriaProteobacteriaAlphaproteobacteriaRhizobialesBeijerinckiaceaeMicrovirga | 0.4599331 | 4 | 2 |
| 43 | BacteriaProteobacteriaAlphaproteobacteriaRhizobialesBeijerinckiaceaePsychroglaciecola | 0.1454334 | 8 | 5 |
| 44 | BacteriaProteobacteriaAlphaproteobacteriaRhizobialesBeijerinckiaceaeunknown_genus | 0.1454334 | 5 | 2 |
| 45 | BacteriaProteobacteriaAlphaproteobacteriaRhizobialesXanthobacteraceaeunknown_genus | 0.20178883 | 5 | 1 |
| 46 | BacteriaProteobacteriaAlphaproteobacteriaSphingomonadalesSphingomonadaceaeEllin6055 | 0.30904597 | 7 | 6 |
| 47 | BacteriaProteobacteriaAlphaproteobacteriaSphingomonadalesSphingomonadaceaeSphingomonas | 0.2908668 | 3 | 3 |
| 48 | BacteriaProteobacteriaGammaproteobacteriaBurkholderialesNitrosomonadaceaeMND1 | 0.22178592 | 3 | 4 |
| 49 | BacteriaProteobacteriaGammaproteobacteriaBurkholderialesTRA3_20unknown_genus | 0.1672484 | 7 | 7 |
| 50 | BacteriaVerrucomicrobiotaVerrucomicrobiaePedosphaeralesPedosphaeraceaeunknown_genus | 0.1672484 | 2 | 4 |
| 51 | EukaryotaMucoromycotaGlomeromycetesDiversisporalesClaroideoglomeraceaeClaroideoglomusGlomeromycotina_sp_ | 1.9160849 | 1 | 8 |
| 52 | EukaryotaMucoromycotaGlomeromycetesDiversisporalesDiversisporaceaeDiversisporaDiversispora_epigaea | 0.20360675 | 6 | 7 |
| 53 | EukaryotaMucoromycotaGlomeromycetesDiversisporalesDiversisporaceaeDiversisporaDiversispora_sp_ | 0.8762362 | 5 | 8 |
| 54 | EukaryotaMucoromycotaGlomeromycetesDiversisporalesDiversisporaceaeDiversisporaDiversispora_spurca | 0.77079695 | 7 | 1 |
| 55 | EukaryotaMucoromycotaGlomeromycetesGlomeralesClaroideoglomeraceaeClaroideoglomusClaroideoglomus_etunicatum | 0.439936 | 2 | 2 |
| 56 | EukaryotaMucoromycotaGlomeromycetesGlomeralesClaroideoglomeraceaeClaroideoglomusClaroideoglomus_lamellosum | 0.42175683 | 6 | 2 |
| 57 | EukaryotaMucoromycotaGlomeromycetesGlomeralesGlomeraceaeFunneliformisFunneliformis_mosseae | 0.17270215 | 4 | 3 |
| 58 | EukaryotaMucoromycotaGlomeromycetesGlomeralesGlomeraceaeGlomusGlomeraceae_sp_ | 0.39085224 | 2 | 5 |
| 59 | EukaryotaMucoromycotaGlomeromycetesGlomeralesGlomeraceaeGlomusGlomeromycotina_sp_ | 1.0143979 | 6 | 8 |
| 60 | EukaryotaMucoromycotaGlomeromycetesGlomeralesGlomeraceaeGlomusGlomus_mycorrhizal_symbiont_of_Marchantia_foliacea | 1.6197644 | 0 | 9 |
| 61 | EukaryotaMucoromycotaGlomeromycetesGlomeralesGlomeraceaeGlomusmetagenome | 2.9632053 | 0 | 1 |
| 62 | EukaryotaMucoromycotaGlomeromycetesGlomeralesGlomeraceaeGlomusunknown_species | 21.155106 | 4 | 2 |
| 63 | EukaryotaMucoromycotaGlomeromycetesGlomeralesGlomeraceaeRhizophagusGlomeromycotina_sp_ | 2.8486764 | 5 | 9 |
| 64 | EukaryotaMucoromycotaGlomeromycetesGlomeralesGlomeraceaeSeptoglomusGlomeromycotina_sp_ | 12.794502 | 3 | 4 |
| 65 | EukaryotaMucoromycotaGlomeromycetesParaglomeralesParaglomeraceaeParaglomusParaglomus_occultum | 1.519779 | 4 | 9 |
| 66 | EukaryotaMucoromycotaGlomeromycetesunknown_orderGlomeraceaeGlomusGlomeraceae_sp_ | 0.3672193 | 4 | 10 |
| 67 | EukaryotaMucoromycotaGlomeromycetesunknown_orderGlomeraceaeGlomusGlomeromycotina_sp_ | 0.858057 | 2 | 4 |
| 68 | EukaryotaMucoromycotaGlomeromycetesunknown_orderGlomeraceaeGlomusunknown_species | 1.1361984 | 2 | 5 |
| 69 | EukaryotaMucoromycotaunknown_classMortierellalesMortierellaceaeMortierellaMortierella_alpina | 1.3288976 | 1 | 5 |
| 70 | FungiAscomycotaDothideomycetesBotryosphaerialesunidentifiedunidentifiedBotryosphaeriales_sp | 0.11816463 | 3 | 10 |
| 71 | FungiAscomycotaDothideomycetesCapnodialesNeodevriesiaceaeNeodevriesiaCapnodiales_sp | 0.12361838 | 2 | 11 |
| 72 | FungiAscomycotaDothideomycetesPleosporalesDidymosphaeriaceaeMontagnulaMontagnula_scabiosae | 1.8560936 | 1 | 5 |
| 73 | FungiAscomycotaDothideomycetesPleosporalesLophiostomataceaeNeovaginatisporaPleosporales_sp | 0.63627106 | 6 | 2 |
| 74 | FungiAscomycotaDothideomycetesPleosporalesPhaeosphaeriaceaeChaetosphaeronemaChaetosphaeronema_sp | 0.59991276 | 1 | 6 |
| 75 | FungiAscomycotaDothideomycetesPleosporalesPleosporaceaeAlternariaAlternaria_chlamydosporigena | 0.9834933 | 1 | 6 |
| 76 | FungiAscomycotaDothideomycetesPleosporalesPleosporaceaeunidentifiedPleosporaceae_sp | 0.11271088 | 1 | 3 |
| 77 | FungiAscomycotaDothideomycetesPleosporalesSporormiaceaeSporormiellaSporormiella_intermedia | 0.17997383 | 0 | 6 |
| 78 | FungiAscomycotaDothideomycetesPleosporalesSporormiaceaeSporormiellaSporormiella_similis | 0.12180047 | 5 | 3 |
| 79 | FungiAscomycotaDothideomycetesunidentifiedBotryosphaeriaceaeunidentifiedDothideomycetes_sp | 0.8253345 | 3 | 11 |
| 80 | FungiAscomycotaDothideomycetesVenturialesSympoventuriaceaeOchroconisOchroconis_sp | 0.11089296 | 4 | 4 |
| 81 | FungiAscomycotaDothideomycetesVenturialesVenturiaceaeVenturiaVenturiaceae_sp | 0.18724549 | 3 | 3 |
| 82 | FungiAscomycotaEurotiomycetesChaetothyrialesTrichomeriaceaeKnufiaMulti_affiliation | 0.10725713 | 1 | 12 |
| 83 | FungiAscomycotaEurotiomycetesOnygenalesOnygenales_fam_Incertae_sedisMalbrancheaMalbranchea_cinnamomea | 0.16543049 | 0 | 7 |
| 84 | FungiAscomycotaEurotiomycetesOnygenalesunidentifiedunidentifiedOnygenales_sp | 0.8944154 | 3 | 4 |
| 85 | FungiAscomycotaLeotiomycetesHelotialesunidentifiedunidentifiedHelotiales_sp | 0.2926847 | 2 | 4 |
| 86 | FungiAscomycotaMulti_affiliationMulti_affiliationDidymellaceaePhomaMulti_affiliation | 0.3654014 | 2 | 6 |
| 87 | FungiAscomycotaPezizomycetesPezizalesPyronemataceaePicoaPicoa_juniperi | 0.2599622 | 0 | 7 |
| 88 | FungiAscomycotaPezizomycetesPezizalesPyronemataceaePseudotricharinaPseudotricharina_intermedia | 0.10180338 | 0 | 6 |
| 89 | FungiAscomycotaPezizomycetesPezizalesPyronemataceaeunidentifiedPyronemataceae_sp | 0.3454043 | 2 | 12 |
| 90 | FungiAscomycotaSordariomycetesHypocrealesBionectriaceaeIjuhyaHypocreales_sp | 0.24360093 | 1 | 13 |
| 91 | FungiAscomycotaSordariomycetesHypocrealesNectriaceaeFusariumFusarium_sp | 0.12725422 | 0 | 13 |
| 92 | FungiAscomycotaSordariomycetesHypocrealesNectriaceaeIlyonectriaIlyonectria_macrodidyma | 0.12361838 | 0 | 4 |
| 93 | FungiAscomycotaSordariomycetesSordarialesChaetomiaceaeHumicolaHumicola_grisea | 0.15997674 | 1 | 5 |
| 94 | FungiAscomycotaSordariomycetesSordarialesLasiosphaeriaceaePodosporaPodospora_sp | 0.17997383 | 1 | 3 |
| 95 | FungiAscomycotaSordariomycetesSordarialesLasiosphaeriaceaeSchizotheciumSchizothecium_carpinicola | 0.11816463 | 1 | 7 |
| 96 | FungiAscomycotaSordariomycetesSordarialesLasiosphaeriaceaeunidentifiedLasiosphaeriaceae_sp | 0.33267888 | 0 | 4 |
| 97 | FungiAscomycotaSordariomycetesunidentifiedChaetomiaceaeParachaetomiumSordariomycetes_sp | 0.39630598 | 0 | 8 |
| 98 | FungiAscomycotaunidentifiedunidentifiedAspergillaceaePenicilliumFungi_sp | 1.805192 | 0 | 14 |
| 99 | FungiAscomycotaunidentifiedunidentifiedChaetomiaceaeHumicolaAscomycota_sp | 0.47447643 | 0 | 6 |

| Senescence | | | | |
| --- | --- | --- | --- | --- |
| Id | Taxa | Relative abundance | Number of interactions | |
|  |  |  | Source | Destination |
| 1 | BacteriaAcidobacteriotaAcidobacteriaeBryobacteralesBryobacteraceaeBryobacter | 0.21572225 | 2 | 0 |
| 2 | BacteriaActinobacteriotaAcidimicrobiiaIMCC26256unknown_familyunknown_genus | 0.15796983 | 6 | 0 |
| 3 | BacteriaActinobacteriotaAcidimicrobiiaMicrotrichalesIamiaceaeIamia | 0.21572225 | 1 | 1 |
| 4 | BacteriaActinobacteriotaAcidimicrobiiaMicrotrichalesunknown_familyunknown_genus | 0.4654165 | 3 | 0 |
| 5 | BacteriaActinobacteriotaAcidimicrobiiaunknown_orderunknown_familyunknown_genus | 0.24799564 | 2 | 0 |
| 6 | BacteriaActinobacteriotaActinobacteria0319_7L14unknown_familyunknown_genus | 0.34481588 | 7 | 0 |
| 7 | BacteriaActinobacteriotaActinobacteriaCorynebacterialesMycobacteriaceaeMycobacterium | 0.16816144 | 2 | 0 |
| 8 | BacteriaActinobacteriotaActinobacteriaFrankialesFrankiaceaeJatrophihabitans | 0.16136703 | 5 | 1 |
| 9 | BacteriaActinobacteriotaActinobacteriaFrankialesGeodermatophilaceaeBlastococcus | 7.6606874 | 16 | 0 |
| 10 | BacteriaActinobacteriotaActinobacteriaFrankialesGeodermatophilaceaeGeodermatophilus | 1.7274766 | 15 | 1 |
| 11 | BacteriaActinobacteriotaActinobacteriaFrankialesGeodermatophilaceaeModestobacter | 0.6522625 | 1 | 1 |
| 12 | BacteriaActinobacteriotaActinobacteriaFrankialesNakamurellaceaeNakamurella | 0.13758662 | 1 | 0 |
| 13 | BacteriaActinobacteriotaActinobacteriaFrankialesSporichthyaceaeunknown_genus | 0.10871042 | 6 | 1 |
| 14 | BacteriaActinobacteriotaActinobacteriaFrankialesunknown_familyunknown_genus | 0.21572225 | 4 | 2 |
| 15 | BacteriaActinobacteriotaActinobacteriaMicrococcalesCellulomonadaceaeCellulomonas | 0.16646284 | 6 | 0 |
| 16 | BacteriaActinobacteriotaActinobacteriaMicrococcalesMicrococcaceaeArthrobacter | 0.12569642 | 7 | 0 |
| 17 | BacteriaActinobacteriotaActinobacteriaMicromonosporalesMicromonosporaceaeActinoplanes | 1.9907596 | 14 | 1 |
| 18 | BacteriaActinobacteriotaActinobacteriaMicromonosporalesMicromonosporaceaeAsanoa | 0.24969426 | 5 | 0 |
| 19 | BacteriaActinobacteriotaActinobacteriaMicromonosporalesMicromonosporaceaeDactylosporangium | 0.113806225 | 0 | 2 |
| 20 | BacteriaActinobacteriotaActinobacteriaMicromonosporalesMicromonosporaceaeKrasilnikovia | 0.67434436 | 4 | 1 |
| 21 | BacteriaActinobacteriotaActinobacteriaMicromonosporalesMicromonosporaceaePhytohabitans | 0.21232505 | 5 | 1 |
| 22 | BacteriaActinobacteriotaActinobacteriaMicromonosporalesMicromonosporaceaeunknown_genus | 0.21572225 | 2 | 1 |
| 23 | BacteriaActinobacteriotaActinobacteriaMicromonosporalesMicromonosporaceaeVirgisporangium | 0.6760429 | 3 | 2 |
| 24 | BacteriaActinobacteriotaActinobacteriaPropionibacterialesNocardioidaceaeNocardioides | 0.4382389 | 2 | 3 |
| 25 | BacteriaActinobacteriotaActinobacteriaPseudonocardialesPseudonocardiaceaeActinophytocola | 0.47221088 | 1 | 2 |
| 26 | BacteriaActinobacteriotaActinobacteriaPseudonocardialesPseudonocardiaceaePseudonocardia | 1.032749 | 5 | 2 |
| 27 | BacteriaActinobacteriotaActinobacteriaStreptomycetalesStreptomycetaceaeStreptomyces | 0.19703764 | 0 | 3 |
| 28 | BacteriaActinobacteriotaActinobacteriaStreptosporangialesThermomonosporaceaeActinomadura | 0.10361462 | 4 | 3 |
| 29 | BacteriaActinobacteriotaMB_A2_108unknown_orderunknown_familyunknown_genus | 0.33292568 | 6 | 1 |
| 30 | BacteriaActinobacteriotaRubrobacteriaRubrobacteralesRubrobacteriaceaeRubrobacter | 4.1496806 | 13 | 3 |
| 31 | BacteriaActinobacteriotaThermoleophiliaGaiellalesGaiellaceaeGaiella | 0.4246501 | 10 | 0 |
| 32 | BacteriaActinobacteriotaThermoleophiliaGaiellalesunknown_familyunknown_genus | 0.73549396 | 12 | 4 |
| 33 | BacteriaActinobacteriotaThermoleophiliaSolirubrobacterales67_14unknown_genus | 2.2455497 | 22 | 0 |
| 34 | BacteriaActinobacteriotaThermoleophiliaSolirubrobacteralesSolirubrobacteraceaeConexibacter | 0.16306563 | 11 | 5 |
| 35 | BacteriaActinobacteriotaThermoleophiliaSolirubrobacteralesSolirubrobacteraceaeParviterribacter | 0.16986004 | 10 | 6 |
| 36 | BacteriaActinobacteriotaThermoleophiliaSolirubrobacteralesSolirubrobacteraceaeSolirubrobacter | 0.9766952 | 9 | 7 |
| 37 | BacteriaEntotheonellaeotaEntotheonelliaEntotheonellalesEntotheonellaceaeCandidatus_Entotheonella | 0.11040902 | 5 | 2 |
| 38 | BacteriaFirmicutesBacilliBacillalesBacillaceaeBacillus | 0.14098383 | 1 | 1 |
| 39 | BacteriaGemmatimonadotaGemmatimonadetesGemmatimonadalesGemmatimonadaceaeGemmatimonas | 0.28366625 | 21 | 1 |
| 40 | BacteriaGemmatimonadotaGemmatimonadetesGemmatimonadalesGemmatimonadaceaeunknown_genus | 0.8391086 | 4 | 2 |
| 41 | BacteriaMyxococcotabacteriap25unknown_orderunknown_familyunknown_genus | 0.16306563 | 1 | 0 |
| 42 | BacteriaProteobacteriaAlphaproteobacteriaAcetobacteralesAcetobacteraceaeCraurococcus_Caldovatus | 0.36010328 | 8 | 8 |
| 43 | BacteriaProteobacteriaAlphaproteobacteriaAcetobacteralesAcetobacteraceaeRoseomonas | 0.13079223 | 3 | 3 |
| 44 | BacteriaProteobacteriaAlphaproteobacteriaAcetobacteralesAcetobacteraceaeunknown_genus | 0.12739503 | 7 | 9 |
| 45 | BacteriaProteobacteriaAlphaproteobacteriaAzospirillalesAzospirillaceaeSkermanella | 0.57922274 | 20 | 2 |
| 46 | BacteriaProteobacteriaAlphaproteobacteriaElsteralesunknown_familyunknown_genus | 0.20213345 | 3 | 4 |
| 47 | BacteriaProteobacteriaAlphaproteobacteriaRhizobialesBeijerinckiaceaeMicrovirga | 1.0361462 | 4 | 3 |
| 48 | BacteriaProteobacteriaAlphaproteobacteriaRhizobialesBeijerinckiaceaeNeo_b11 | 0.13418943 | 0 | 1 |
| 49 | BacteriaProteobacteriaAlphaproteobacteriaRhizobialesBeijerinckiaceaePsychroglaciecola | 0.26837885 | 19 | 3 |
| 50 | BacteriaProteobacteriaAlphaproteobacteriaRhizobialesBeijerinckiaceaeunknown_genus | 0.27177605 | 2 | 5 |
| 51 | BacteriaProteobacteriaAlphaproteobacteriaRhizobialesXanthobacteraceaeunknown_genus | 0.20213345 | 9 | 1 |
| 52 | BacteriaProteobacteriaAlphaproteobacteriaSphingomonadalesSphingomonadaceaeEllin6055 | 0.33632287 | 18 | 4 |
| 53 | BacteriaProteobacteriaAlphaproteobacteriaSphingomonadalesSphingomonadaceaeSphingomonas | 0.27347466 | 17 | 5 |
| 54 | BacteriaProteobacteriaAlphaproteobacteriaTistrellalesGeminicoccaceaeCandidatus_Alysiosphaera | 0.21911944 | 8 | 2 |
| 55 | BacteriaProteobacteriaGammaproteobacteriaBurkholderialesNitrosomonadaceaeMND1 | 0.15966843 | 6 | 10 |
| 56 | BacteriaProteobacteriaGammaproteobacteriaBurkholderialesTRA3_20unknown_genus | 0.16646284 | 1 | 6 |
| 57 | EukaryotaMucoromycotaGlomeromycetesDiversisporalesClaroideoglomeraceaeClaroideoglomusGlomeromycotina_sp_ | 1.1448567 | 7 | 3 |
| 58 | EukaryotaMucoromycotaGlomeromycetesDiversisporalesDiversisporaceaeDiversisporaDiversispora_epigaea | 0.22421524 | 1 | 4 |
| 59 | EukaryotaMucoromycotaGlomeromycetesDiversisporalesDiversisporaceaeDiversisporaDiversispora_sp_ | 0.4705123 | 16 | 6 |
| 60 | EukaryotaMucoromycotaGlomeromycetesDiversisporalesDiversisporaceaeDiversisporaDiversispora_spurca | 0.25309145 | 3 | 3 |
| 61 | EukaryotaMucoromycotaGlomeromycetesGlomeralesClaroideoglomeraceaeClaroideoglomusClaroideoglomus_etunicatum | 0.8017394 | 5 | 11 |
| 62 | EukaryotaMucoromycotaGlomeromycetesGlomeralesClaroideoglomeraceaeClaroideoglomusClaroideoglomus_lamellosum | 0.67944014 | 3 | 4 |
| 63 | EukaryotaMucoromycotaGlomeromycetesGlomeralesGlomeraceaeFunneliformisFunneliformis_mosseae | 0.13758662 | 2 | 5 |
| 64 | EukaryotaMucoromycotaGlomeromycetesGlomeralesGlomeraceaeGlomusGlomeraceae_sp_ | 0.31593966 | 1 | 6 |
| 65 | EukaryotaMucoromycotaGlomeromycetesGlomeralesGlomeraceaeGlomusGlomeromycotina_sp_ | 1.2977307 | 6 | 4 |
| 66 | EukaryotaMucoromycotaGlomeromycetesGlomeralesGlomeraceaeGlomusGlomus_mycorrhizal_symbiont_of_Marchantia_foliacea | 3.0795624 | 15 | 7 |
| 67 | EukaryotaMucoromycotaGlomeromycetesGlomeralesGlomeraceaeGlomusmetagenome | 6.388436 | 14 | 8 |
| 68 | EukaryotaMucoromycotaGlomeromycetesGlomeralesGlomeraceaeGlomusunknown_species | 5.0142684 | 5 | 5 |
| 69 | EukaryotaMucoromycotaGlomeromycetesGlomeralesGlomeraceaeRhizophagusGlomeromycotina_sp_ | 0.8221226 | 2 | 4 |
| 70 | EukaryotaMucoromycotaGlomeromycetesGlomeralesGlomeraceaeSeptoglomusGlomeromycotina_sp_ | 9.457807 | 0 | 5 |
| 71 | EukaryotaMucoromycotaGlomeromycetesParaglomeralesParaglomeraceaeParaglomusParaglomus_occultum | 1.3418943 | 4 | 6 |
| 72 | EukaryotaMucoromycotaGlomeromycetesunknown_orderGlomeraceaeGlomusGlomeromycotina_sp_ | 1.632355 | 3 | 7 |
| 73 | EukaryotaMucoromycotaGlomeromycetesunknown_orderGlomeraceaeGlomusGlomus_sp_ | 0.16306563 | 0 | 7 |
| 74 | EukaryotaMucoromycotaGlomeromycetesunknown_orderGlomeraceaeGlomusunknown_species | 0.20213345 | 1 | 5 |
| 75 | EukaryotaMucoromycotaunknown_classMortierellalesMortierellaceaeMortierellaMortierella_alpina | 0.87477916 | 13 | 9 |
| 76 | EukaryotaMucoromycotaunknown_classMortierellalesParaglomeraceaeParaglomusPhaseoleae_environmental_sample | 0.113806225 | 2 | 8 |
| 77 | FungiAscomycotaDothideomycetesBotryosphaerialesunidentifiedunidentifiedBotryosphaeriales_sp | 0.25818726 | 0 | 7 |
| 78 | FungiAscomycotaDothideomycetesPleosporalesLophiostomataceaeNeovaginatisporaPleosporales_sp | 0.33292568 | 12 | 10 |
| 79 | FungiAscomycotaDothideomycetesPleosporalesPhaeosphaeriaceaeChaetosphaeronemaChaetosphaeronema_sp | 0.47221088 | 4 | 12 |
| 80 | FungiAscomycotaDothideomycetesPleosporalesPleosporaceaeAlternariaAlternaria_chlamydosporigena | 1.2060063 | 2 | 4 |
| 81 | FungiAscomycotaDothideomycetesPleosporalesPleosporaceaeAlternariaAlternaria_subcucurbitae | 0.10361462 | 0 | 1 |
| 82 | FungiAscomycotaDothideomycetesPleosporalesSporormiaceaeSporormiellaSporormiella_similis | 0.16306563 | 0 | 2 |
| 83 | FungiAscomycotaDothideomycetesunidentifiedBotryosphaeriaceaeunidentifiedDothideomycetes_sp | 1.5779997 | 3 | 13 |
| 84 | FungiAscomycotaDothideomycetesVenturialesVenturiaceaeVenturiaVenturiaceae_sp | 3.230738 | 11 | 11 |
| 85 | FungiAscomycotaEurotiomycetesChaetothyrialesTrichomeriaceaeKnufiaKnufia_perforans | 0.5520451 | 1 | 5 |
| 86 | FungiAscomycotaEurotiomycetesEurotialesAspergillaceaePenicilliumMulti_affiliation | 0.5197717 | 2 | 14 |
| 87 | FungiAscomycotaEurotiomycetesEurotialesAspergillaceaePenicilliumPenicillium_aurantiogriseum | 0.14947683 | 1 | 15 |
| 88 | FungiAscomycotaEurotiomycetesEurotialesAspergillaceaePenicilliumPenicillium_novae_zeelandiae | 2.4850523 | 10 | 12 |
| 89 | FungiAscomycotaEurotiomycetesOnygenalesOnygenales_fam_Incertae_sedisMalbrancheaMalbranchea_cinnamomea | 0.31424105 | 9 | 13 |
| 90 | FungiAscomycotaEurotiomycetesOnygenalesunidentifiedunidentifiedOnygenales_sp | 3.4209812 | 8 | 14 |
| 91 | FungiAscomycotaLeotiomycetesHelotialesHamatocanthoscyphaceaeHamatocanthoscyphaMulti_affiliation | 0.15796983 | 0 | 6 |
| 92 | FungiAscomycotaLeotiomycetesHelotialesunidentifiedunidentifiedHelotiales_sp | 0.21911944 | 1 | 9 |
| 93 | FungiAscomycotaMulti_affiliationMulti_affiliationDidymellaceaePhomaMulti_affiliation | 0.3533089 | 7 | 15 |
| 94 | FungiAscomycotaPezizomycetesPezizalesPyronemataceaeunidentifiedPyronemataceae_sp | 0.11040902 | 0 | 16 |
| 95 | FungiAscomycotaSordariomycetesHypocrealesBionectriaceaeIjuhyaHypocreales_sp | 0.100217424 | 0 | 6 |
| 96 | FungiAscomycotaSordariomycetesHypocrealesNectriaceaeFusariumFusarium_sp | 0.18854465 | 6 | 16 |
| 97 | FungiAscomycotaSordariomycetesHypocrealesNectriaceaeIlyonectriaIlyonectria_macrodidyma | 0.30574808 | 5 | 17 |
| 98 | FungiAscomycotaSordariomycetesSordarialesChaetomiaceaeBotryotrichumMulti_affiliation | 0.5809213 | 4 | 18 |
| 99 | FungiAscomycotaSordariomycetesSordarialesChaetomiaceaeHumicolaHumicola_grisea | 0.7541786 | 3 | 19 |
| 100 | FungiAscomycotaSordariomycetesSordarialesLasiosphaeriaceaeApiosordariaSordariales_sp | 0.27857044 | 0 | 2 |
| 101 | FungiAscomycotaSordariomycetesSordarialesLasiosphaeriaceaeunidentifiedLasiosphaeriaceae_sp | 0.19024324 | 0 | 10 |
| 102 | FungiAscomycotaunidentifiedunidentifiedAspergillaceaePenicilliumFungi_sp | 4.305952 | 2 | 20 |
| 103 | FungiAscomycotaunidentifiedunidentifiedChaetomiaceaeHumicolaAscomycota_sp | 0.19364044 | 1 | 21 |
| 104 | FungiBasidiomycotaTremellomycetesFilobasidialesPiskurozymaceaeSolicoccozymaSolicoccozyma_aeria | 0.13418943 | 0 | 22 |
